# Supplementary material for: Inferring spatial and signaling relationships between cells from single cell transcriptomic data
Source: Nat Commun. 2020 Apr 29;11:2084. doi: 10.1038/s41467-020-15968-5 (PMC7190659; doi:10.1038/s41467-020-15968-5)
Supplement: Supplementary file 1 — Supplementary Information [file 41467_2020_15968_MOESM1_ESM.pdf]

Supplementary Information

Inferring spatial and signaling relationships between cells from single cell transcriptomic data

Zixuan Cang[1,3] and Qing Nie[1,2,3*]

[1] Department of Mathematics, [2] Department of Developmental and Cell Biology, and [3] The NSF-Simons Center for Multiscale Cell Fate Research, University of California, Irvine, CA 92697, USA

Supplementary Methods

Supplementary Figures 1-35

* To whom correspondence should be addressed. Email: qnie@uci.edu.

Supplementary Methods

Contents

1 SpaOTsc model **2**
1.1 Optimal transport 2
1.1.1 The unbalanced structured optimal transport 2
1.1.2 Wasserstein distances 3
1.1.3 Numerical approximation 4
1.2 Acceleration by approximations 5
1.2.1 Approximation of Wasserstein distance with landmark points 5
1.2.2 Approximation of geodesic distances 6
1.3 Mapping between spatial data and single-cell data 6
1.4 Spatial distance between single cells 7
1.5 Clustering of spatial gene expression patterns 7
1.6 Weight kernels 8
1.7 Intercellular gene-gene regulatory information flow 8
1.7.1 Mutual information 9
1.7.2 Partial information decomposition 10
1.7.3 Information flow network construction 11
1.8 Spatial distance for intercellular signaling 12
1.8.1 Feature importance in random forest 12
1.8.2 Inference of spatial signaling range 13
1.9 Cell-cell communication 14
1.9.1 Optimal transport cell-cell communication 14
1.9.2 Additional spatial distance constraint in optimal transport cell-cell communication 16
1.9.3 Communication index 17

2 Assessment of model performance **17**
2.1 Cross-validation of spatial gene expression prediction . . . 17
2.2 Impact of using unbalanced and structured optimal transport 18
2.3 Intercellular gene-gene information flow 19
2.4 Effect of number of background genes used in intercellular process analyses 19

3 Genes used in intercellular process analyses **19**
3.1 Cell-cell communication in drosophila embryo 20
3.2 Cell-cell communication in zebrafish embryo 21
3.3 Spatial distance of signaling in drosophila embryo 21

1 SpaOTsc model

1.1 Optimal transport

Optimal transport theory finds an optimal (under predefined cost) transportation plans from a distribution to another.

1.1.1 The unbalanced structured optimal transport

Consider a problem where the two distributions to be mapped are discretely represented by vectors $\boldsymbol{\omega}_1 \in \mathbb{R}^n$ and $\boldsymbol{\omega}_2 \in \mathbb{R}^m$. Let $\mathbf{M} \in \mathbb{R}^{n\times m}$, $\mathbf{A}_1 \in \mathbb{R}^{n\times n}$, and $\mathbf{A}_2 \in \mathbb{R}^{m\times m}$ be dissimilarity measurements for bins across two distributions and within each distribution. A transport plan can be represented by $\boldsymbol{\gamma} \in \mathbb{R}_+^{n\times m}$ which specifies how much mass from each bin of one distribution is transported to each bin of the other distribution indicating a mapping between the two datasets. Measurements of different aspects are defined to assess the quality of transport plans. The major one is the transport cost defined as

$$E_{\text{transport}} =< \boldsymbol{\gamma}, \mathbf{M} >_{\text{F}} . \quad (1)$$

Since scRNA-seq data and spatial data are usually not from the same samples and there are cells lost in experiments, we should allow unbalanced transport, i.e. the transport desinations of a bin do not need to add up to the same mass of this bin. This is realized by using a penalty term

for the unbalance in $\boldsymbol{\gamma}$ instead of strict constraint.[6] Kullback-Leibler (KL) divergence is used to measure the divergence of two measures

$$\mathrm{KL}(\mathbf{h}|\mathbf{p}) = \sum_i \mathbf{h}_i \log\left(\frac{\mathbf{h}_i}{\mathbf{p}_i}\right) - \mathbf{h}_i + \mathbf{p}_i. \tag{2}$$

The penalty term for unbalance in $\boldsymbol{\gamma}$ is defined based on KL divergence

$$E_{\text{unbalance}} = \mathrm{KL}(\boldsymbol{\gamma}\mathbf{1}^m|\boldsymbol{\omega}_1) + \mathrm{KL}(\boldsymbol{\gamma}^T\mathbf{1}^n|\boldsymbol{\omega}_2). \tag{3}$$

The Gromov-Wasserstein (GW) distance measures difference between metric spaces.[16] In other words, it focuses on how well the intra-dataset relationship is preserved by the mapping. The GW loss is defined as

$$E_{\text{structure}} = \sum_{i,j,k,\ell} L(\mathbf{A}_1(i,k), \mathbf{A}_2(j,\ell))\boldsymbol{\gamma}_{i,j}\boldsymbol{\gamma}_{k,\ell}. \tag{4}$$

A linear combination of Eq. 1 and 4 has been proposed for structured data.[24]. In practice, a regularization term is often used to accelerate convergence of the algorithm. The entropy regularization term is defined as

$$H(\boldsymbol{\gamma}) = \sum_{i,j} \boldsymbol{\gamma}_{i,j} \log(\boldsymbol{\gamma}_{i,j}). \tag{5}$$

The three loss terms defined above assemble to the total loss for finding the mapping between scRNA-seq data and spatial data.

$$\underset{\boldsymbol{\gamma}\in\mathbb{R}_+^{n\times m}}{\arg\min}(1-\alpha)E_{\text{transport}} + \rho E_{\text{unbalance}} + \alpha E_{\text{structure}}. \tag{6}$$

1.1.2 Wasserstein distances

The optimal transport framework naturally provides a measurement for difference between distributions. We use this distance to measure (1) distance between spatial distributions of individual cells as indicator of spatial distance between cells and (2) distance between spatial gene expression patterns. In the later application, we can both quantify this distance on the original geometry or directly on the single cell data paired with cell-cell spatial distances. The Wasserstein distance is defined as

$$d_{\mathrm{W}}(\boldsymbol{\omega}_1, \boldsymbol{\omega}_2, \mathbf{M}) = \min_{\boldsymbol{\gamma}\in\mathbb{R}_+^{n\times m}} < \boldsymbol{\gamma}, \mathbf{M} >_{\mathrm{F}} . \tag{7}$$

It is often solved with an entropy term defined in Eq. 5. The outcome of this regularized version is called Sinkhorn distance which is an approximation to Wasserstein distance. Note that when the cost matrix satisfies to be a metric, this distance is also a metric. In SpaOTsc, Wasserstein distance is used to compute distances between single cells based on distance matrix of spatial locations resulting in a distance matrix for single cells satisfying to be a metric. This distance matrix for single cells is further used as the cost matrix when characterizing differences between genes thus resulting in a metric for spatial expression patterns of genes.

1.1.3 Numerical approximation

We focus on the case $L = L_2$ which results in a quadratic optimization problem and follow the optimization scheme given in [24]. The transport part and structure part in Eq. 6 can be written as

$$f_\ell(\boldsymbol{\gamma}) =< (1-\alpha)\mathbf{M} + \alpha\mathcal{L}\otimes\boldsymbol{\gamma}, \boldsymbol{\gamma} >_{\mathrm{F}}, \tag{8}$$

where $\mathcal{L}_{i,j,k,\ell} = L\big(\mathbf{A}_1(i,j), \mathbf{A}_2(k,\ell)\big)$ and $\mathcal{L}\otimes\boldsymbol{\gamma} = \Big(\sum_{k,\ell}\mathcal{L}_{i,j,k,\ell}\boldsymbol{\gamma}_{k,\ell}\Big)_{i,j}$. The proposition 1 in [16] suggests that if L can be written as

$$L(a,b) = f_1(a) + f_2(b) - h_1(a)h_2(b)$$

for some functions f_1, f_2, h_1, h_2, then $\mathcal{L}\otimes\boldsymbol{\gamma}$ can be decomposed as

$$\mathcal{L}\otimes\boldsymbol{\gamma} = c_{\mathbf{A}_1,\mathbf{A}_2} - h_1(\mathbf{A}_1)\boldsymbol{\gamma}h_2(\mathbf{A}_2)^T,$$

where $c_{\mathbf{A}_1,\mathbf{A}_2}$ is independent of $\boldsymbol{\gamma}$. This property provides an efficient way of calculating $\mathcal{L}\otimes\boldsymbol{\gamma}$. When the quadratic loss $L_2(a,b) = \frac{1}{2}(a-b)^2$ is used, the functions can be constructed as

$$\begin{aligned} f_1(x) &= f_2(x) = x^2/2, \\ h_1(x) &= h_2(x) = x. \end{aligned} \tag{9}$$

Proposition 2 in [16] gives the gradient of the Gromov-Wasserstein part of the loss function,

$$\nabla_{\boldsymbol{\gamma}} < \mathcal{L}\otimes\boldsymbol{\gamma}, \boldsymbol{\gamma} >= 2\mathcal{L}\otimes\boldsymbol{\gamma}.$$

So the gradient of the loss function in Eq. 8 can be computed as

$$\nabla_{\boldsymbol{\gamma}} f_\ell = (1-\alpha)\mathbf{M} + 2\alpha\mathcal{L}\otimes\boldsymbol{\gamma}. \tag{10}$$

The Frank-Wolfe algorithm is used to find local minima. The direction-finding subproblem in the algorithm can be regarded as an optimal transport problem

$$\mathbf{p}_k = \underset{\mathbf{p}\in\Gamma}{\arg\min} < (1-\alpha)\mathbf{M} + 2\alpha\mathcal{L}\otimes\boldsymbol{\gamma}_{k-1}, \mathbf{p} > +\rho E_{\text{unbalance}}. \tag{11}$$

Here we substitute the usual strict restriction of mass conservation by the soft penalty term $E_{\text{unbalance}}$ to allow unbalance transport. This problem is solved following [6]. Then a step size τ is determined which minimizes $f_\ell(\boldsymbol{\gamma}_{k-1} + \tau(\mathbf{p}_k - \boldsymbol{\gamma}_{k-1}))$ subject to $\tau \in [0,1]$ where the function can be written as

$$\begin{aligned}
&f_\ell(\boldsymbol{\gamma}_{k-1} + \tau(\mathbf{p}_k - \boldsymbol{\gamma}_{k-1})) \\
&= < (1-\alpha)\mathbf{M} + \alpha c_{\mathbf{A}_1,\mathbf{A}_2} - \alpha\mathbf{A}_1(\boldsymbol{\gamma}_{k-1} + \tau(\mathbf{p}_k - \boldsymbol{\gamma}_{k-1}))\mathbf{A}_2^T, \boldsymbol{\gamma}_{k-1} + \tau(\mathbf{p}_k - \boldsymbol{\gamma}_{k-1}) > \\
&= < (1-\alpha)\mathbf{M} + \alpha c_{\mathbf{A}_1,\mathbf{A}_2}, \boldsymbol{\gamma}_{k-1} > \\
&+\tau < (1-\alpha)\mathbf{M} + \alpha c_{\mathbf{A}_1,\mathbf{A}_2} - 2\alpha\mathbf{A}_1\boldsymbol{\gamma}_{k-1}\mathbf{A}_2^T, \mathbf{p}_k - \boldsymbol{\gamma}_{k-1} > \\
&+\tau^2 < -\alpha\mathbf{A}_1(\mathbf{p}_k - \boldsymbol{\gamma}_{k-1})\mathbf{A}_2^T, \mathbf{p}_k - \boldsymbol{\gamma}_{k-1} > .
\end{aligned}$$

Some terms are combined assuming $\mathbf{A}_1$ and $\mathbf{A}_2$ are symmetric. Let τ^* be the minimizer satisfying $0 \leq \tau^* \leq 1$. The new transport plan is updated as $\boldsymbol{\gamma}_k = \tau^*\mathbf{p}_k + (1-\tau^*)\boldsymbol{\gamma}_{k-1}$.

1.2 Acceleration by approximations

1.2.1 Approximation of Wasserstein distance with landmark points

When computing spatial distance between individual cells, we accelerate the computation by approximating the spatial geometry with fewer landmark points. We follow a method proposed to approximate the topology induced by point clouds.[7]. When the spatial locations are regarded as a point cloud, the algorithm initializes by randomly selecting a point from the point cloud as a landmark point. We call the points in the point cloud that are not landmark points the candidate points. At each step, we first compute the shortest distance from each candidate point to the collection of landmark points. Then a candidate point with the largest shortest distance is selected as a landmark point. This process is iterated until the target number of landmark points is achieved. The information carried by

the non-landmark points are assigned to the nearest landmark points. Optimal transport is computed on landmark points as an approximation for the distance computed on the original point cloud.

1.2.2 Approximation of geodesic distances

Tissues often have nontrivial shapes which motivates the use of geodesic distances for spatial locations. To efficiently approximate the geodesic distances between spatial locations of *in situ* data, we construct a graph based on k-nearest neighbors and compute the lengths of shortest paths. Given a collection of points with their spatial coordinates, we first build a k-nearest neighbor graph based on the Euclidean distance which is also used as edge weights. If there are separate connected subgraphs, an edge is added between two closest points from each pair of such subgraphs. Now we have a path-connected graph with nodes representing the points. The shortest path length between each two nodes on this graph is computed to approximate geodesic distances between points. This approximate geodesic distance matrix is denoted by $\mathbf{D}_{\text{geo}}(\mathbf{X}, k)$ where k is the number of nearest neighbors used and $\mathbf{X}$ is the point cloud.

1.3 Mapping between spatial data and single-cell data

Consider a single cell dataset with n cells and a spatial dataset with m spatial locations. To initialize the computation of optimal transport, we first need to construct the distance matrices $\mathbf{M}, \mathbf{A}_1, \mathbf{A}_2$ listed at the beginning of Section 1.1.1. To compute the similarity of two binary vectors $\mathbf{x}$ and $\mathbf{y}$, we use Matthews correlation coefficient. Spearman's rank-order correlation coefficient is used for numerical valued vectors. Both correlation coefficients ranges from -1 for totally opposite relation to 1 for perfect match. When number of genes of moderate, we directly apply these correlation coefficients to the gene expression vectors. A pre-processing is carried out to reduce the dimensionality when a large number of genes are involved. Within a dataset, for example the scRNA-seq data, we use principal component analysis to map the data onto a lower dimensional space to robustly quantify differences between individual cells. When comparing across datasets, we use canonical correlation analysis to map both datasets onto a common low dimensional space following [4]. The correla-

tion coefficients are transformed into a distance measurement by applying a exponential function $(\mathrm{e}^{-c} - \mathrm{e}^{-1})/(\mathrm{e} - \mathrm{e}^{-1})$.

Once we have the distance matrices, we solve the optimization problem in Eq. 6. The optimal transport plan $\boldsymbol{\gamma}^* \in \mathbb{R}_+^{n\times m}$ is a matrix serving as mapping between the data sets (n single cells and m spatial locations). The entry $\boldsymbol{\gamma}^*_{i,j}$ depicts the likelihood of cell i originated from location j. This map is used to reconstruct spatial gene expressions by $\hat{\boldsymbol{\gamma}^*}^{\mathrm{T}} * \mathrm{g}$ where $\mathrm{g} \in \mathbb{R}^{n,n_g}$ is a matrix recording gene expressions in single-cell data and $\hat{\boldsymbol{\gamma}^*}$ is column normalized (each column sums to 1) version of $\boldsymbol{\gamma}^*$. The location of the maximum entry of each row of $\boldsymbol{\gamma}^*$ delivers a prediction of spatial origin of the single cell corresponding to this row.

1.4 Spatial distance between single cells

Using the cost matrix constructed in the previous section, we obtain a transport matrix $\boldsymbol{\gamma}^* \in \mathbb{R}_+^{n\times m}$ connecting scRNAseq data with n cells and spatial data with m locations. Let $\mathbf{D}_{\mathrm{spa}} \in \mathbb{R}_+^{m\times m}$ be a distance matrix between spatial locations. The spatial distance between two single cells i and j is indicated by the Wasserstein distance:

$$\hat{\mathbf{D}}_{\mathrm{sc}}(i,j) = d_{\mathrm{W}}(\bar{\boldsymbol{\gamma}^*}_i, \bar{\boldsymbol{\gamma}^*}_j, \mathbf{D}_{\mathrm{spa}}), \tag{12}$$

where $\bar{\boldsymbol{\gamma}^*}_i$ is the ith row of $\boldsymbol{\gamma}^*$ normalized to have a sum of 1.

The spatial distance matrix of single cells $\hat{\mathbf{D}}_{\mathrm{sc}}$ directly leads to several applications. (1) Spatially meaningful low dimensional visualization of single-cell data is obtained by feeding the $\hat{\mathbf{D}}_{\mathrm{sc}}$ to dimension reduction algorithms that can work with distance matrices, such as tSNE and UMAP. (2) Spatially localized sub-populations of cells are identified by performing sub-clustering based on $\hat{\mathbf{D}}_{\mathrm{sc}}$ within cell clusters determined in previous single-cell data analysis.

1.5 Clustering of spatial gene expression patterns

In this section, we discuss the quantitative comparison of gene expression patterns using optimal transport. Since the setup of computational optimal transport does not explicitly require geometry, we can directly compare using single cell data in addition to comparing using predicted spatial expressions. In the case of comparing directly in single cell data, let $\mathbf{g}_a, \mathbf{g}_b \in \mathbb{R}^n$

be the vectors of expression of these two genes in individual cells, and $\hat{\mathbf{D}}_{\rm sc} \in \mathbb{R}^{n\times n}$ be the spatial distance between in the single cells. The difference of spatial gene expression pattern is quantified by $d_{\rm W}(\mathbf{g}_a, \mathbf{g}_b, \hat{\mathbf{D}}_{\rm sc})$, the Wasserstein distance defined in Eq. 7. The inputs $\mathbf{g}_a$ and $\mathbf{g}_b$ are normalized first to have sum of 1. The case of comparing spatial expression in the original geometry is similar with the inputs being the normalized expression across spatial locations and the distance matrix explicitly constructed using geodesic distance in the original geometry. A python implementation [23] of louvain clustering algorithm [2] with the RBConfiguration method [18] is used to cluster the genes based on a neighborhood graph built with the quantified spatial pattern differences.

1.6 Weight kernels

Thresholding is often used in analysis of genes or spatial relationships. To avoid the unstability caused by hard thresholding, we use continuous kernel functions to assign weights that reflects spatial relations. These smooth weight functions have shown to outperform hard cutoffs in modeling protein structures [28]. We list two commonly used weight kernels here. The exponential kernel and the generalized Lorentz kernel are defined as

$$\begin{aligned} \phi_{\rm Exp}(\|x_i - x_j\|; \eta, \nu) &= {\rm e}^{-(\|x_i - x_j\|/\eta)^\nu}, \\ \phi_{\rm Lorentz}(\|x_i - x_j\|; \eta, \nu) &= \frac{1}{1 + (\|x_i - x_j\|/\eta)^\nu}, \end{aligned} \tag{13}$$

where η is a scaling parameter analogous to the cutoff distance in hard thresholding approaches and the parameter ν controls the steepness of decay near η. These kernels can also be used to substitute hard thresholding of significance as $\phi(1/s; 1/\eta, \nu)$ with s being a signal which is considered significant when exceeds the scaling parameter η. The weight kernels will be used throughout the following analysis methods.

1.7 Intercellular gene-gene regulatory information flow

Consider two variables depicting the expression of a gene in the spatial neighborhoods of cells and the expression of a gene in the cells. We quantify how much information about the second variable is provided by the first.

We call this intercellular gene-gene information flow in the sense of information theory. In the following sections, we call the genes corresponding to the variables the source gene and the target gene respectively. Note that the source gene and the target gene can be the same, in which case, we are measuring a non-linear analog of spatial autocorrelation.

1.7.1 Mutual information

The entropy of a random variable X measures how much information it carries and can be written as

$$H(X) = \sum_{x \in X} -p(x) \log_2(p(x)). \tag{14}$$

The base 2 of the logarithm results in the units of shannons. There are other choices that lead to other information units. The conditional entropy can be defined in a similar manner,

$$H(X|Y) = \sum_{x \in X, y \in Y} -p(x,y) \log_2 \left(\frac{p(x,y)}{p(y)} \right). \tag{15}$$

Conditional entropy measures how much information is carried in X that cannot be told by observing Y. Also, the joint entropy can be written as

$$H(X,Y) = \sum_{x \in X, y \in Y} -p(x,y) \log_2(p(x,y)). \tag{16}$$

Then, the mutual information between X and Y measures how much information does observing one variable provide about the other can be computed by

$$I(X;Y) = \sum_{x \in X, y \in Y} -p(x,y) \log_2 \left(\frac{p(x,y)}{p(x)p(y)} \right). \tag{17}$$

The mutual information can also be equivalently expressed by the entropy terms,

$$\begin{aligned} I(X;Y) &= H(X) + H(Y) - H(X,Y) \\ &= H(X,Y) - H(X|Y) - H(Y|X) \\ &= H(X) - H(X|Y) \\ &= H(Y) - H(Y|X). \end{aligned} \tag{18}$$

1.7.2 Partial information decomposition

We are interested in the case with three variables X, Y, Z in this application. Specifically, we seek to inquire how much unique information is provided about Z by observing Y when X is already observed. Partial information decomposition (PID) is proposed by Paul and Randall [27]. When considering X and Y together, we can measure how much information is provided about Z by X and Y using mutual information $I(Z; X, Y)$. PID states that the information provided by X and Y about Z can be classified into 4 categories: (1-2) uniquely provided by X or Y that the other variable can not provide (unique information), (3) jointly provided by X and Y that they can not provide alone (synergy), and (4) both X and Y can provide alone (redundancy). PID seeks to decompose $I(Z; X, Y)$ into these four parts to decipher the detailed information flow among the three variables. The decomposition can be expressed as

$$\begin{aligned} I(Z; X, Y) = {} & \mathrm{Unq}_X(Z; Y) + \mathrm{Unq}_Y(Z; X) \\ & + \mathrm{Syn}(Z; X, Y) + \mathrm{Rdn}(Z; X, Y). \end{aligned} \tag{19}$$

We briefly go over one formulation of the decomposition by [27] which starts by computing redundancy. The mutual information between Z and X can be decomposed by each outcome of Z as

$$I(Z; X) = \sum_{z \in Z} p(z) I_{\mathrm{spec}}(Z = z; X). \tag{20}$$

The term I_{spec} is called the specific information,

$$I_{\mathrm{spec}}(Z = z; X) = \sum_{x \in X} p(x|z) \left(\log_2 \left(\frac{1}{p(z)} \right) - \log_2 \left(\frac{1}{p(z|x)} \right) \right). \tag{21}$$

Then the redundancy $\mathrm{Rdn}(Z; X, Y)$ is quantified by

$$I_{\min}(Z; X, Y) = \sum_{z \in Z} p(z) \min_{S \in \{X, Y\}} I_{\mathrm{spec}}(Z = z; S). \tag{22}$$

This construction relies on the perspective that the minimum information provided by the sources reflects the common information and thus redundancy. Once redundancy is computed, the unique information can be computed as

$$\mathrm{Unq}_X(Z; Y) = I(Z; Y) - \mathrm{Rdn}(Z; X, Y). \tag{23}$$

The implementation ($\mathrm{PID\_WB}$) in the python package discrete information theory (dit) is used [11].

1.7.3 Information flow network construction

When applying PID to the inference of inter-cellular gene talks, we consider a target gene $G_{\rm tar}$, a source gene in the spatial neighborhoods $\widetilde{G}_{\rm src}$, and a collection of background genes $\mathcal{G}$ that are related to $G_{\rm tar}$. The observation of $\widetilde{G}$ under a spatial distance η can be constructed with the weight kernels defined in Eq. 13,

$$\widetilde{\mathbf{g}}_i = \sum_j \mathbf{W}_{i,j} * \mathbf{g}_i / \sum_j \mathbf{W}_{i,j}, \tag{24}$$

where $\mathbf{W}_{i,j} = \phi_(\hat{\mathbf{D}}_{\rm sc}(i,j); \eta, \nu)$ for some positive ν. It is desirable to directly compute $\mathrm{Unq}_{\mathcal{G}}(G_{\rm tar}; \widetilde{G}_{\rm src})$ to assess the unique information about the target gene provided by the expression of source gene in spatial neighborhoods upon knowing the background genes. However, this decomposition can quickly become computationally intractable as the lattice becomes huge even with a moderate size of $\mathcal{G}$. Fortunately, for a target gene and a set of candidate source genes, we are able to compare the source genes relatively. In other words, we can use the average unique information in the three variable case instead,

$$u(G_{\rm src}, G_{\rm tar}, \eta) = \sum_{G \in \mathcal{G}} \mathrm{Unq}_G(G_{\rm tar}; \widetilde{G}_{\rm src}). \tag{25}$$

As these analysis are carried out in the discrete setting of information theory, we pre-processed the gene expression levels into discrete bins using the implementation of Bayesian blocks [22] provided in the Astropy package [20, 17].

The score defined in Eq. 25 requires the computation of $O(n^3)$ partial information decomposition which could become very time consuming when the number of genes considered is large. We use a weighted spatial correlation coefficient [5] as a pre-screening of significant pairs before performing PID. The weighted spatial correlation coefficient is defined as

$$R_c = \left(\frac{\mathbf{x} - \mu_x}{\sigma_x}\right)^T \mathbf{W} \left(\frac{\mathbf{y} - \mu_y}{\sigma_y}\right), \tag{26}$$

where μ and σ are mean value and standard deviation and $\mathbf{W}$ is a spatial weight matrix satisfying $\mathbf{W}_{ii} = 0$ and $\sum_{ij} \mathbf{W}_{ij} = 1$.

1.8 Spatial distance for intercellular signaling

Intercellular signaling follows a general mechanism involving mainly ligands, receptors and downstream genes. Based on this explicit mechanism, we aim at inferring an effective spatial distance for known signaling. To this end, we use ensemble of trees models which are interpretable and are able to address the signaling mechanism.

1.8.1 Feature importance in random forest

Random forest is an ensemble of independently trained decision trees [3]. It is assumed that individual trees make different mistakes and their consensus may achieve better performance. In practice, random forest is usually an efficient close match to state-of-the-art results in tasks with moderate sample and feature size. Here, we briefly review the feature importance measurement in random forest regression models. A decision tree is a tree with each node equipped with a criteria associated to a feature. When a sample is fed to the tree, it flows from the root to a leaf which then gives the predicted target value for this sample. An important score used to build a decision tree, the impurity score of a node can be calculated as

$$\mathrm{NI} = \frac{1}{N}\sum_{i=1}^{N} |\mathbf{y}_i - \bar{\mathbf{y}}|, \tag{27}$$

where $\mathbf{y}_i$ are the target values of the training samples that go through this node and $\bar{\mathbf{y}}$ is the average of these values. The process of training a decision tree can be roughly understood as choosing features to add new nodes to the tree which mostly decrease the impurity. At the same time, impurity scores can also be used to assess the importance of features. The relative importance of a feature in a tree can be quantified by

$$\mathrm{FI} = \sum_{i\in\mathcal{N}} \left(\mathrm{NI}_i - \sum_{j\in\mathcal{C}_i} \frac{N_j}{N_i}\mathrm{NI}_j \right), \tag{28}$$

where $\mathcal{N}$ is the index set for nodes associated to this feature, $\mathcal{C}_i$ is the index set of for the child nodes of node i, and N_k is the number of training samples that flow through node j. Then, the normalized importance of

feature i in a tree can be computed as

$$\mathrm{NFI}_i = \mathrm{FI}_i \Big/ \sum_{j=1}^{N_\mathrm{f}} \mathrm{FI}_j, \tag{29}$$

where N_f is the number of features in the tree. Then, the importance of a feature across the whole forest can be simply quantified by an average of its normalized feature importance in all the trees that assembles the forest. The random forest implementation in scikit-learn is used.[15]

1.8.2 Inference of spatial signaling range

Here, we build random forest regression models that predict the expression of a target gene A (a downstream gene in a signaling) based on a collection of genes within the same cell that are closely related to A and the expression of gene B (the ligand in this signaling) in the neighborhood. The samples are weighted by the expression level of the receptor(s) involved in the signaling. Specifically, the sample weights are constructed as

$$\mathbf{w}_i = \phi(R_i; \eta_r, \nu), \tag{30}$$

where η_r is a scaling parameter deciding whether the receptor is significantly expressed and ν is a negative integer. Given a spatial distance η, we first construct a weight matrix $\mathbf{W} \in \mathbb{R}^{n\times n}$ for the cells,

$$\mathbf{W}_{i,j} = \begin{cases} \phi(\hat{\mathbf{D}}(i,j); \eta, \nu), \ i \neq j, \\ 0, \ i = j \end{cases} \tag{31}$$

where $\hat{\mathbf{D}}(i,j)$ is the inferred spatial distance between cell i and j and ϕ is the kernel function defined in Eq. 13 with a positive ν. Let $\bar{\mathbf{W}}$ be the row normalized matrix of $\mathbf{W}$, i.e. $\bar{\mathbf{W}}_{i,j} = \mathbf{W}_{i,j} / \sum_j \mathbf{W}_{i,j}$. The vector representing the expression of gene B in each cells' neighborhood can be approximated as

$$\mathbf{x}_\mathrm{nb} = \bar{\mathbf{W}}\mathbf{x}, \tag{32}$$

where $\mathbf{x}$ is the column vector for expression of gene B. Similar to Eq. 25, the random forest model is built with additional features depicting the expression of a collection of background genes $\mathcal{G}$. The signaling strength under this spatial distance is then indicated by the average of this feature

importance score over a collection of downstream genes. Computing this signaling strength for a sequence of spatial distances provides us a hint of the effective range of this particular signaling.

1.9 Cell-cell communication

With the inferred spatial cell-cell distance, we can analyze cell-cell communication by examining the intercellular signaling in a spatial setting.

1.9.1 Optimal transport cell-cell communication

For an intercellular signaling where we know the ligand, receptor(s) and downstream genes, we interpret the signaling process as a transport problem where the ligands are transported from their source distribution (described by the expression level of the ligand gene) to a destination distribution (depicted by the expression level of receptors and the downstream genes). Intuitively, if a cell highly expresses the receptor genes and the expression of downstream genes are consistent with the known up/down regulation relationship, this cell is likely to be a target cell in the signaling.

For the source distribution in the transport problem, we simply use the expression level of ligands,

$$\boldsymbol{\omega}_{\mathrm{L}}(i) = \mathbf{L}_i / \sum_i \mathbf{L}_i, \tag{33}$$

where $\mathbf{L}_i$ is the expression level of the ligand gene in cell i. A scoring is defined to quantify how consistent is the expression pattern of downstream genes according to the known up/down regulation relationships

$$\boldsymbol{\beta}_j = \frac{1}{n_{\mathrm{D}}} \sum_{\ell} \phi\big(\mathbf{D}_{j,\ell}; \eta_\ell, \nu_\ell\big), \tag{34}$$

where n_{D} is the number of downstream genes, $\mathbf{D}_{j,\ell}$ is the ℓth downstream gene in cell j, η_ℓ is a scaling parameter for soft thresholding of gene expressions, and ν_ℓ is positive for down-regulated genes and negative for up-regulated genes. A bigger value for $|\nu_\ell|$ is used if a steeper boundary is desired in the weight kernel defined in Eq. 13. If a strict consistency is

desired, we can use multiplicative penalty

$$\hat{\boldsymbol{\beta}}_j = \left(\prod_{\ell} \phi(\mathbf{D}_{j,\ell}; \eta_\ell, \nu_\ell) \right)^{1/n_{\mathrm{D}}}. \tag{35}$$

The destination distribution for the signaling is then described by

$$\boldsymbol{\omega}_{\mathrm{D}}(j) = \mathbf{R}_j * \boldsymbol{\beta}_j / (\sum_j \mathbf{R}_j * \boldsymbol{\beta}_j). \tag{36}$$

Another working assumption is that it is more likely for ligands to get to their spatial neighbors than far away locations. This makes it natural to use our spatial cell-cell distance matrix $\hat{\mathbf{D}}_{\mathrm{sc}}$ as our cost matrix for the optimal transport problem. Finally, we set up an optimal transport problem:

$$\begin{aligned} \underset{\boldsymbol{\gamma} \in \mathbb{R}_+^{n \times n}}{\arg\min} \Big[& < \boldsymbol{\gamma}, \hat{\mathbf{D}}_{\mathrm{sc}} >_{\mathrm{F}} \\ & + \rho \left(\mathrm{KL}(\boldsymbol{\gamma} \mathbf{1}^n | \boldsymbol{\omega}_{\mathrm{L}}) + \mathrm{KL}(\boldsymbol{\gamma}^T \mathbf{1}^n | \boldsymbol{\omega}_{\mathrm{D}}) \right) \\ & + \lambda \sum_{i,j} \boldsymbol{\gamma}_{i,j} \log(\boldsymbol{\gamma}_{i,j}) \Big] \end{aligned} \tag{37}$$

where KL is the KL divergence. Note that the last term (entropy regularization) in Eq. 37 was used to accelerate computation in previous applications. In this signaling application, this entropy term accounts for the stochasticity of ligand diffusion, i.e., it penalizes the case that all ligands from a cell go to the same closest cell. The optimal transport plan $\boldsymbol{\gamma}^*_{\mathrm{S}}(i,j)$ is interpreted as a scoring for cell j receiving signal from cell i.

There are also cases where spatial distance for certain signaling is available by applying inference methods or from knowledge. In this case, we can tweak the transport cost in Eq. 37 to account for this constraint,

$$\bar{\mathbf{D}}_{\mathrm{sc}}(i,j) = \begin{cases} \hat{\mathbf{D}}_{\mathrm{sc}}(i,j), \text{ if} \hat{\mathbf{D}}_{\mathrm{sc}}(i,j) \leq D_{\mathrm{S}}, \\ \infty, \text{otherwise}, \end{cases} \tag{38}$$

where D_{S} is the given spatial distance of signaling. The extremely high cost of transporting between cells with distance farther than the signaling distance prevents the mapping between these cells.

Compared to methods that analyze cell pairs independently, this approach naturally addresses an important property of ligand-receptor signaling, i.e. it is likely for a cell releasing signaling ligands to have shorter signaling range if it is surrounded by more consumers of this ligands (cells highly expressing receptors and show consistent behavior for downstream genes). Further more, this approach implicitly takes into account the spatial constraint and closely resembles the inferred cell-cell communication networks that are explicitly constrained by inferred spatial distance for signaling.

1.9.2 Additional spatial distance constraint in optimal transport cell-cell communication

When prior knowledge about the effective range of a ligand is available, we can further constrain the optimal transport inference of signaling. We implement the extra spatial distance constraint by setting the transport cost between cells that exceeds this distance to an excessively large number. As a result, long connections will be eliminated and additional short connections might merge. We discuss the effect of adding a spatial distance constraint below.

On the single-cell level, for illustration, we consider a simple system with three cells: a signal sender cell (cell 1) and two signal receiver cells (cell 2 and cell 3) (see Supplementary Fig. 22). Cell 3 is more likely a receiver cell than cell 2 (in other words, cell 3 has a greater mass than cell 2 as destinations in the optimal transport setup) but the distance between cell 3 and cell 1, d(cell 1, cell 3) is greater than d(cell 1, cell 2). Without the spatial constraint, we would identify a stronger connection between cell 1 and cell 3 than between cell 1 and cell 2 due to the conservation of mass. If a spatial constraint that is between d(cell 1, cell 3) and d(cell 1, cell 2) is applied, we would observe no mass going from cell 1 to cell 3 (thus the previous strong connection vanishes) but more mass going from cell 1 to cell 2 (the emergence of a shorter connection). In the optimal transport formulation, the transportation cost (a large cost if moving much mass along long distance) and the mass conservation violation (a penalty for unbalanced mass transport) are the two penalty terms in the objective function to be minimized. When a spatial constraint is applied, a connection whose distance is longer than this constraint will induce a huge penalty in the transportation cost while the elimination of this connection might lead to

the emergence of new short connections which only induces a moderate penalty in mass conservation violation. Therefore, we may observe new connections when a spatial constraint is applied.

On the cluster level, there are also new connections of visually long distances when the spatial constraint is added (Supplementary Fig. 18). This is because we used a geometric average to represent the location of clusters. So there are clusters that are adjacent in space while their cluster centers are visually distant (e.g. cluster 4.1 and cluster 3.1 in Supplementary Fig. 18b). As a result, we may observe a new connection between clusters of visually long distance contributed by cell pairs of short distance located near the interfaces where the two clusters meet.

1.9.3 Communication index

We also give another simple approach by adding a spatial weight term to a scoring index similar to the one introduced in [26]. The scoring index quantifying the likelihood of cell i signaling to cell j is constructed as

$$\mathbf{S}_{i,j} = \phi(\hat{\mathbf{D}}_{\mathrm{sc}}(i,j);\eta_d,\nu_d)\frac{(\boldsymbol{\alpha}_{i,j}\boldsymbol{\beta}_j)/(\boldsymbol{\alpha}_{i,j}+\boldsymbol{\beta}_j)}{\sum_k(\boldsymbol{\alpha}_{i,k}\boldsymbol{\beta}_k)/(\boldsymbol{\alpha}_{i,k}+\boldsymbol{\beta}_k)}, \tag{39}$$

where $\alpha_{i,j}$ measures the significance of ligand-(transporter)-receptor,

$$\boldsymbol{\alpha}_{i,j} = \phi(1/(\mathbf{L}_i\mathbf{R}_j\mathbf{T}_i);1/\eta_\alpha,\nu_\alpha) \tag{40}$$

where $\mathbf{L}_i$ is the ligand in cell i, $\mathbf{T}_i$ is the sum of transporter for this ligand in cell i, $\mathbf{R}_j$ is the corresponding receptor in cell j. The index $\boldsymbol{\beta}_j$ measuring the consistency of downstream genes in cell j is the same as constructed in Eq. 34 and Eq. 35.

2 Assessment of model performance

2.1 Cross-validation of spatial gene expression prediction

We evaluated the quality of the derived mapping between spatial data and scRNA-seq data by assessing the accuracy in spatial gene expression prediction. Specifically, we did a leave-one-out cross-validation. The spatial

	Drosophila embryo			
	AUC (b.)	R_s (b.)	RMSE (c.)	R_s (c.)
SpaOTsc	0.876	0.495	0.225	0.424
DistMap	0.818	0.409	0.303	0.339
Achim	0.847	0.451	0.278	0.379
	Zebrafish embryo (1)		Zebrafish embryo (2)	
	AUC (b.)	R_s (b.)	AUC (b.)	R_s (b.)
SpaOTsc	0.952	0.681	0.936	0.657
DistMap	0.939	0.663	0.926	0.642
Achim	0.929	0.645	0.887	0.579
Seurat (v1)	0.942	0.667	0.911	0.619

Table 1: Performance comparison on three pairs of datasets. AUC: area-under-curve for receiver operating characteristic curve. R_s: Spearman's correlation coefficient. RMSE: root-mean-squared-error. b.: binarized spatial data. c.: numerical spatial data. Drosophila embryo: spatial data and scRNA-seq data both from ref. [13]. Zebrafish embryo (1): spatial data and scRNA-seq data both from ref. [21]. Zebrafish embryo (2): spatial data from ref. [21] and scRNA-seq data from ref. [25]. Methods compared to: DistMap (ref. [13]), Achim (ref. [1], Seurat (ref. [21]).

expression of a gene is predicted using the rest of the spatial data and the entire scRNA-seq data. Several metrics were used depending on the type of spatial data (binary or continuous). Several established methods designated for this particular problem were considered: DistMap [13], Achim [1], and Seurat [21].

2.2 Impact of using unbalanced and structured optimal transport

We alter the parameters α and ρ in Eq. 6 to assess the usefulness of combining structured transport and allowing unbalanced transport. The parameter α ranges from 0 meaning regular optimal transport without structured information to 1 meaning the pure Gromov-Wasserstein distance. When the parameter ρ takes the value of $+\infty$, this becomes balanced optimal transport and the balance constraint is relieved as ρ decreases. The exper-

iment results suggest that both relaxing the balance constraint and adding the structured loss help improve the accuracy (**Supplementary Fig. 1-4**).

2.3 Intercellular gene-gene information flow

The concept of intercellular gene-gene information flow is in the perspective of information, i.e. how helpful observing a gene in the environment is for inferring the a gene in the cell. The pairs of genes we identify may be connected via various mechanisms of intercellular communications. Here we test our approach on known signalings. We anticipate that the information flow from a ligand gene to its target gene should be relatively higher. While we are not able to cover all target genes in a signaling, the score should still be higher on average if we examine a large unbiased collection of genes. We analyzed Dpp and Wg signaling and observed expected patterns (**Supplementary Fig. 29**).

2.4 Effect of number of background genes used in intercellular process analyses

Ideally, we would like to iterate over all the genes when computing the intercellular gene information flows or to use all other genes as background genes in the machine learning models when inferring distance for cell-cell signaling. However, this can become computationally intractable especially in the case of partial information decomposition. Therefore, we rank the genes by their correlation to the target gene and choose the top subset as the background genes. We altered the number of top genes used and the experiments shows that a moderate size of 50 genes is enough to capture the key features (**Supplementary Fig. 24-28**).

3 Genes used in intercellular process analyses

The the analysis of cell-cell communication for drosophila embryo, we considered Dpp and Wg signaling. We list here the genes involved.

3.1 Cell-cell communication in drosophila embryo

Dpp signaling:

- Ligands: *dpp*
- Receptors: *tkv, put, sax*
- Downstream genes (up-regulated): *Ance, zen2, zen, ush, Doc1, Doc2, Doc3, tup, egr, kay, peb, C15*

The downstream genes are taken from the supplementary table (S1 Dataset) of [8]. The genes marked as "Y" in the column called "Dpp targets" and are also present in scRNA-seq data are considered.
Wg signaling:

- Ligands: *wg, Wnt5.*
- Receptors: *fz.*
- Downstream genes (up-regulated): *CycD, Jra, en.*
- Downstream genes (down-regulated): *dpp.*

EGF signaling:

- Ligands: *spi.*
- Ligand transporters: *rho.*
- Receptors: *Egfr.*
- Downstream genes (up-regulated): *aos, kek1, sty, rho, vn.*

The genes *CycD* and *Jra* are taken from Kegg database [12] (Wnt signaling pathway - Drosophila melanogaster). Two other genes *en*[10] and *dpp*[29] are identified from literature. The genes for EGF signaling are taken from [14].

3.2 Cell-cell communication in zebrafish embryo

Wnt signaling:

- Ligands: *wnt8a, wnt8-2, wnt11.*
- Receptors: *fzd7a, fzd8a, fzd8b, fzd10.*
- Downstream genes (up-regulated): *mycb, jun, fosl1a, ccnd1, ccnd2a, ppardb, tcf7l2, tcf7l1a, lef1, dvl2.*
- Downstream genes (down-regulated): *gsk3aa.*

BMP signaling:

- Ligands: *bmp2b, bmp4, bmp7a.*
- Receptors: *bmpr1aa, bmpr1ab, bmpr1ba, bmpr1bb, bmpr2a, bmpr2b.*
- Downstream genes (up-regulated): *id1, id2a, id2b, id3, id4, smad1, smad5, smad9.*

FGF signaling:

- Ligands: *fgf8a, fgf17.*
- Receptors: *fgfr4.*
- Downstream genes (up-regulated): *spry4.*

The genes of Wnt and BMP signaling are taken from Kegg database [12] (Wnt signaling pathway - Danio rerio and TGF-beta signaling pathway - Danio rerio). The genes for FGF signaling are taken from [9].

3.3 Spatial distance of signaling in drosophila embryo

We filtered out downstream genes whose variances are too small in the inference of signaling distance using random forest model. The following downstream genes are considered:

- Dpp signaling: *Ance, ush, Doc1, Doc2, Doc3, tup, egr*
- Wg signaling: *CycD, Jra, en, dpp, Ubx*

In Wg signaling analysis, there is an additional gene *Ubx*[19] that was not considered in cell-cell communication because it is unknown whether it is up- or down-regulated by Wg.

References

[1] K. Achim, J.-B. Pettit, L. R. Saraiva, D. Gavriouchkina, T. Larsson, D. Arendt, and J. C. Marioni. High-throughput spatial mapping of single-cell rna-seq data to tissue of origin. *Nature biotechnology*, 33(5):503, 2015.

[2] V. D. Blondel, J.-L. Guillaume, R. Lambiotte, and E. Lefebvre. Fast unfolding of communities in large networks. *Journal of statistical mechanics: theory and experiment*, 2008(10):P10008, 2008.

[3] L. Breiman. Random forests. *Machine learning*, 45(1):5–32, 2001.

[4] A. Butler, P. Hoffman, P. Smibert, E. Papalexi, and R. Satija. Integrating single-cell transcriptomic data across different conditions, technologies, and species. *Nature biotechnology*, 36(5):411, 2018.

[5] Y. Chen. A new methodology of spatial cross-correlation analysis. *PloS one*, 10(5):e0126158, 2015.

[6] L. Chizat, G. Peyré, B. Schmitzer, and F.-X. Vialard. Scaling algorithms for unbalanced optimal transport problems. *Mathematics of Computation*, 87(314):2563–2609, 2018.

[7] V. De Silva and G. E. Carlsson. Topological estimation using witness complexes. *SPBG*, 4:157–166, 2004.

[8] L. Deignan, M. T. Pinheiro, C. Sutcliffe, A. Saunders, S. G. Wilcockson, L. A. Zeef, I. J. Donaldson, and H. L. Ashe. Regulation of the bmp signaling-responsive transcriptional network in the drosophila embryo. *PLoS genetics*, 12(7):e1006164, 2016.

[9] M. Fürthauer, J. Van Celst, C. Thisse, and B. Thisse. Fgf signalling controls the dorsoventral patterning of the zebrafish embryo. *Development*, 131(12):2853–2864, 2004.

[10] J. E. Hooper. Distinct pathways for autocrine and paracrine wingless signalling indrosophila embryos. *Nature*, 372(6505):461, 1994.

[11] R. G. James, C. J. Ellison, and J. P. Crutchfield. dit: a Python package for discrete information theory. *The Journal of Open Source Software*, 3(25):738, 2018.

[12] M. Kanehisa, Y. Sato, M. Furumichi, K. Morishima, and M. Tanabe. New approach for understanding genome variations in kegg. *Nucleic acids research*, 47(D1):D590–D595, 2018.

[13] N. Karaiskos, P. Wahle, J. Alles, A. Boltengagen, S. Ayoub, C. Kipar, C. Kocks, N. Rajewsky, and R. P. Zinzen. The drosophila embryo at single-cell transcriptome resolution. *Science*, 358(6360):194–199, 2017.

[14] J. Lusk, V. Lam, and N. Tolwinski. Epidermal growth factor pathway signaling in drosophila embryogenesis: tools for understanding cancer. *Cancers*, 9(2):16, 2017.

[15] F. Pedregosa, G. Varoquaux, A. Gramfort, V. Michel, B. Thirion, O. Grisel, M. Blondel, P. Prettenhofer, R. Weiss, V. Dubourg, J. Vanderplas, A. Passos, D. Cournapeau, M. Brucher, M. Perrot, and E. Duchesnay. Scikit-learn: Machine learning in Python. *Journal of Machine Learning Research*, 12:2825–2830, 2011.

[16] G. Peyré, M. Cuturi, and J. Solomon. Gromov-wasserstein averaging of kernel and distance matrices. In *International Conference on Machine Learning*, pages 2664–2672, 2016.

[17] A. Price-Whelan, B. Sipőcz, H. Günther, P. Lim, S. Crawford, S. Conseil, D. Shupe, M. Craig, N. Dencheva, A. Ginsburg, et al. The astropy project: building an open-science project and status of the v2. 0 core package. *The Astronomical Journal*, 156(3):123, 2018.

[18] J. Reichardt and S. Bornholdt. Partitioning and modularity of graphs with arbitrary degree distribution. *Physical Review E*, 76(1):015102, 2007.

[19] J. Riese, X. Yu, A. Munnerlyn, S. Eresh, S.-C. Hsu, R. Grosschedl, and M. Bienz. Lef-1, a nuclear factor coordinating signaling inputs from wingless and decapentaplegic. *Cell*, 88(6):777–787, 1997.

[20] T. P. Robitaille, E. J. Tollerud, P. Greenfield, M. Droettboom, E. Bray, T. Aldcroft, M. Davis, A. Ginsburg, A. M. Price-Whelan, W. E. Kerzendorf, et al. Astropy: A community python package for astronomy. *Astronomy & Astrophysics*, 558:A33, 2013.

[21] R. Satija, J. A. Farrell, D. Gennert, A. F. Schier, and A. Regev. Spatial reconstruction of single-cell gene expression data. *Nature biotechnology*, 33(5):495, 2015.

[22] J. D. Scargle, J. P. Norris, B. Jackson, and J. Chiang. Studies in astronomical time series analysis. vi. bayesian block representations. *The Astrophysical Journal*, 764(2):167, 2013.

[23] V. Traag. louvain-igraph. `https://github.com/vtraag/louvain-igraph`, 2016.

[24] T. Vayer, L. Chapel, R. Flamary, R. Tavenard, and N. Courty. Optimal transport for structured data. *arXiv preprint arXiv:1805.09114*, 2018.

[25] D. E. Wagner, C. Weinreb, Z. M. Collins, J. A. Briggs, S. G. Megason, and A. M. Klein. Single-cell mapping of gene expression landscapes and lineage in the zebrafish embryo. *Science*, 360(6392):981–987, 2018.

[26] S. Wang, M. Karikomi, A. L. MacLean, and Q. Nie. Cell lineage and communication network inference via optimization for single-cell transcriptomics. 03 2019.

[27] P. L. Williams and R. D. Beer. Nonnegative decomposition of multivariate information. *arXiv preprint arXiv:1004.2515*, 2010.

[28] K. Xia, K. Opron, and G.-W. Wei. Multiscale multiphysics and multidomain modelsflexibility and rigidity. *The Journal of chemical physics*, 139(19):11B614_1, 2013.

[29] X. Yang, M. Van Beest, H. Clevers, T. Jones, D. Hursh, and M. Mortin. decapentaplegic is a direct target of dtcf repression in the drosophila visceral mesoderm. *Development*, 127(17):3695–3702, 2000.

Supplementary Figures

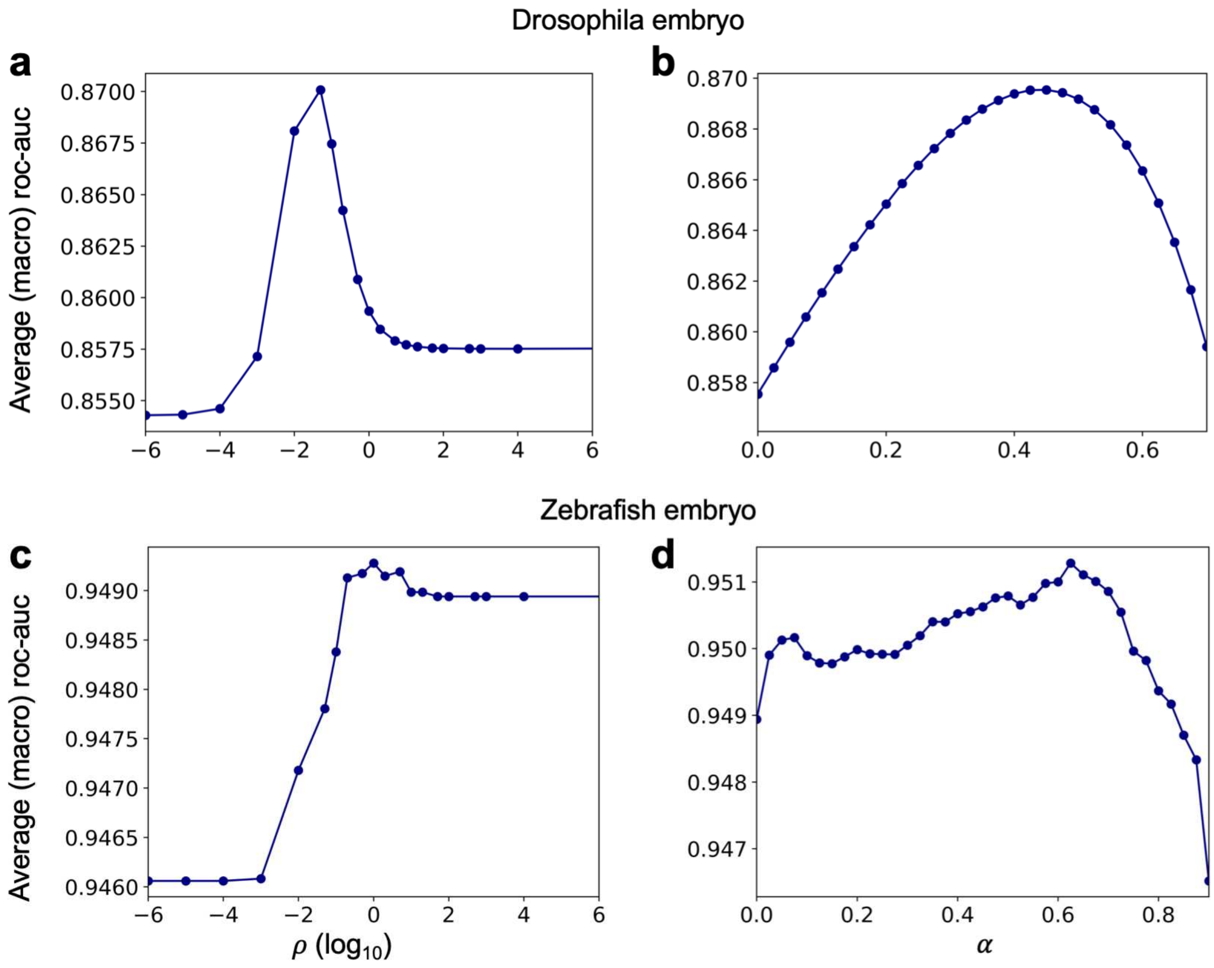

Supplementary Figure 1

Performance of structured and unbalanced optimal transport.

The parameter α is the weight for structured transport penalty and ρ is the weight for the penalty term of unbalanced transport. (**a**) The auc of leave-one-out cross validation of drosophila embryo data with varying weights for unbalance penalty ρ while the structured penalty weight α=0. (**b**) The auc of leave-one-out cross validation of drosophila embryo data with varying weights for structured penalty α while only balanced transport is allowed ($\rho = \infty$). (**c, d**) Same plots as (a) and (b) for zebrafish embryo data. Both scRNA-seq data and spatial data are taken from (Satija, R. *et al.,* Nature Biotech., 2015).

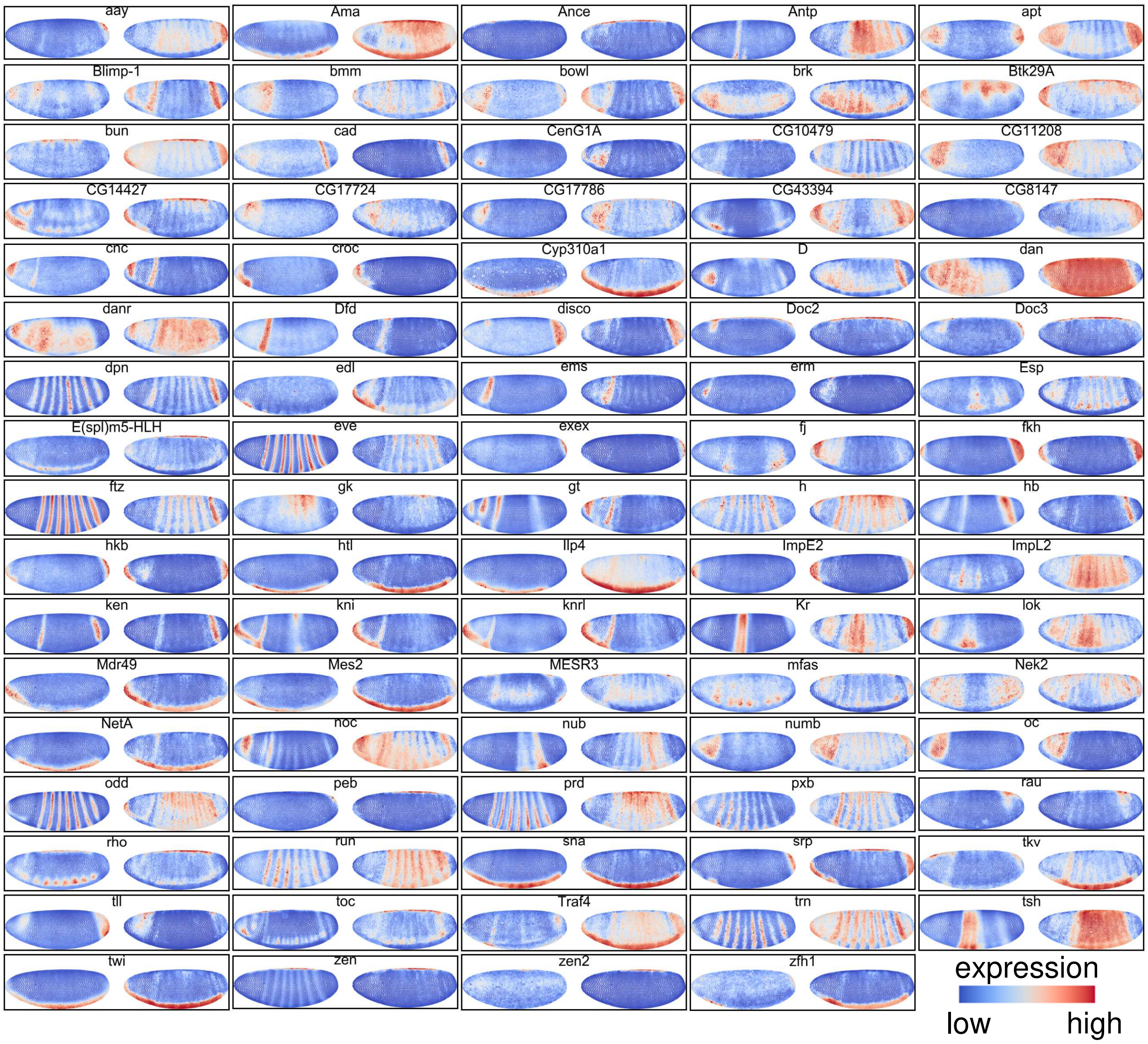

Supplementary Figure 2

Leave-one-out cross validation on drosophila embryo data.

The ground truth (left columns) and prediction (right columns) of spatial gene expression in the leave-one-out cross validation of the 84 genes in spatial data of drosophila embryo.

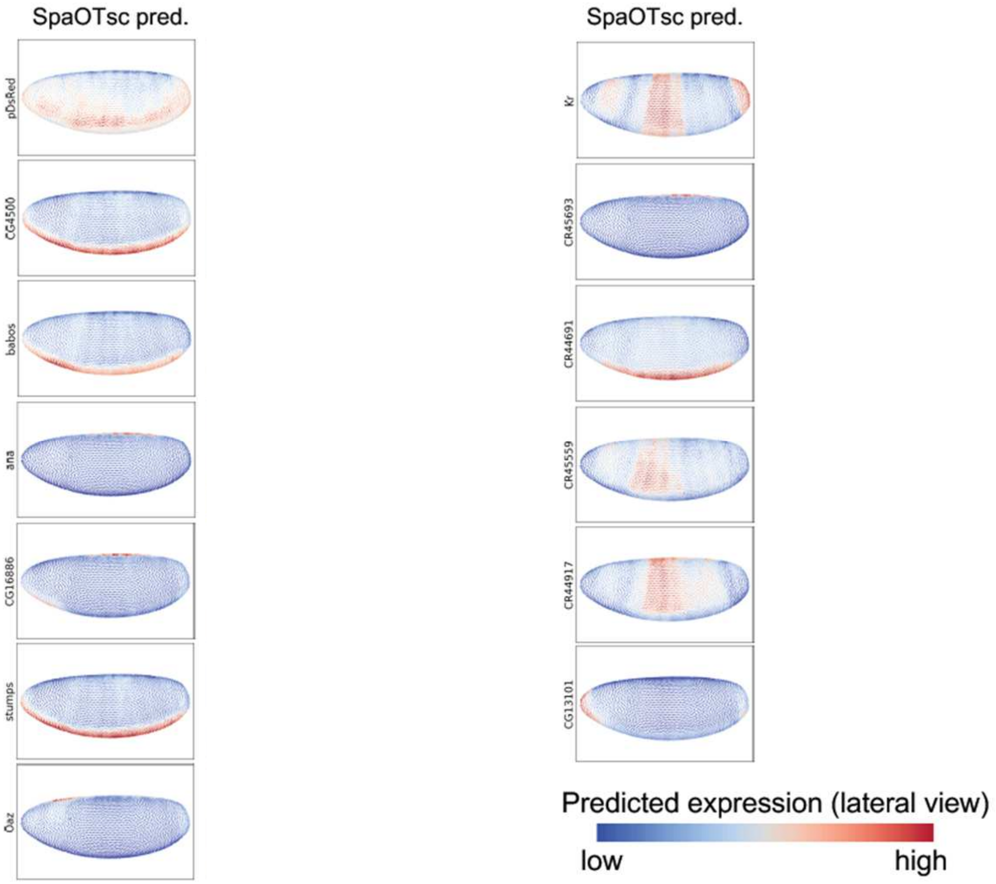

Supplementary Figure 3

Additional drosophila embryo gene expression prediction.

The experimental data for these genes can be found in the Supplementary Figure S4 from Karaikos, Niko, et al. “The Drosophila embryo at single-cell transcriptome resolution.” Science 358.6360 (2017): 194-199. The gene m4 is skipped since it is absent in the scRNA-seq data. The prediction is based on the scRNA-seq data, and the spatial data which does not include the above genes.

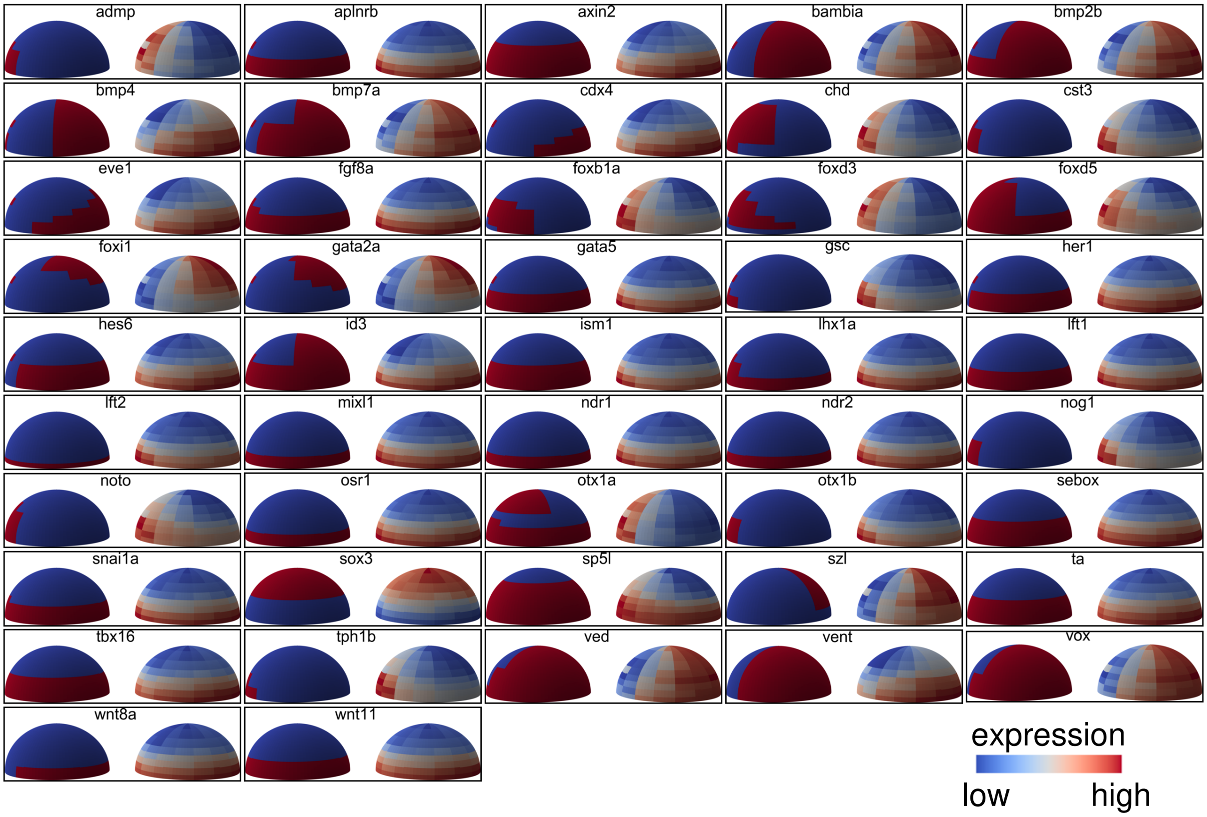

Supplementary Figure 4

Leave-one-out cross-validation zebrafish embryo data.

The ground truth (left columns) and prediction (right columns) of spatial gene expression in the leave-one-out cross-validation of the 47 genes in spatial data of zebrafish embryo. Both scRNA-seq data and spatial data are taken from (Satija, R. *et al.,* Nature Biotech., 2015).

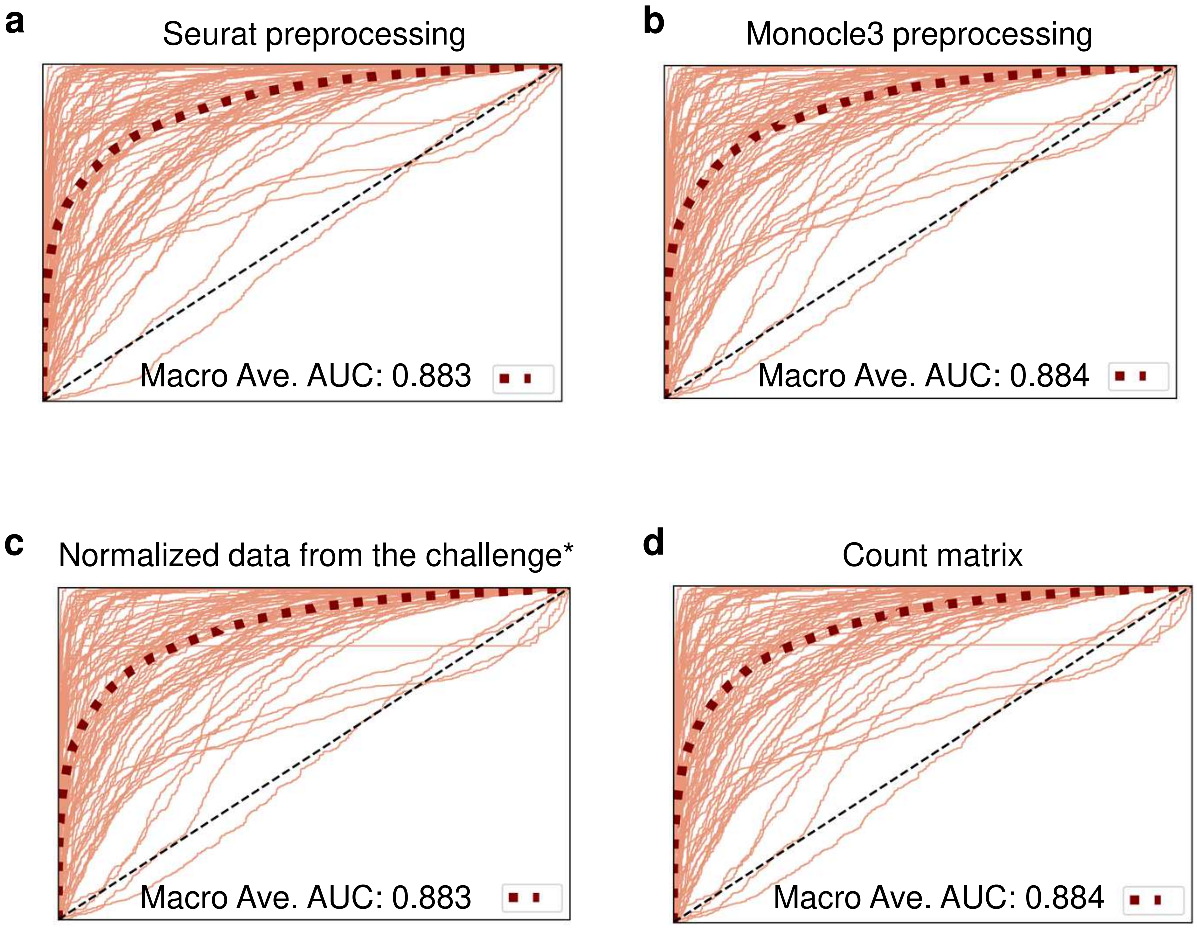

Supplementary Figure 5

Prediction accuracy with different preprocessing procedures.

The similarity between scRNA-seq data and the spatial data is quantified by Spearman's correlation coefficient of the common genes. The numerical spatial data is used. The similarity within the scRNA-seq data is quantified by the Spearman's correlation coefficient of first 50 principal components. *Normalized data downloaded from the DREAM Single Cell Transcriptomics Challenge (Synapse ID: syn15665609).

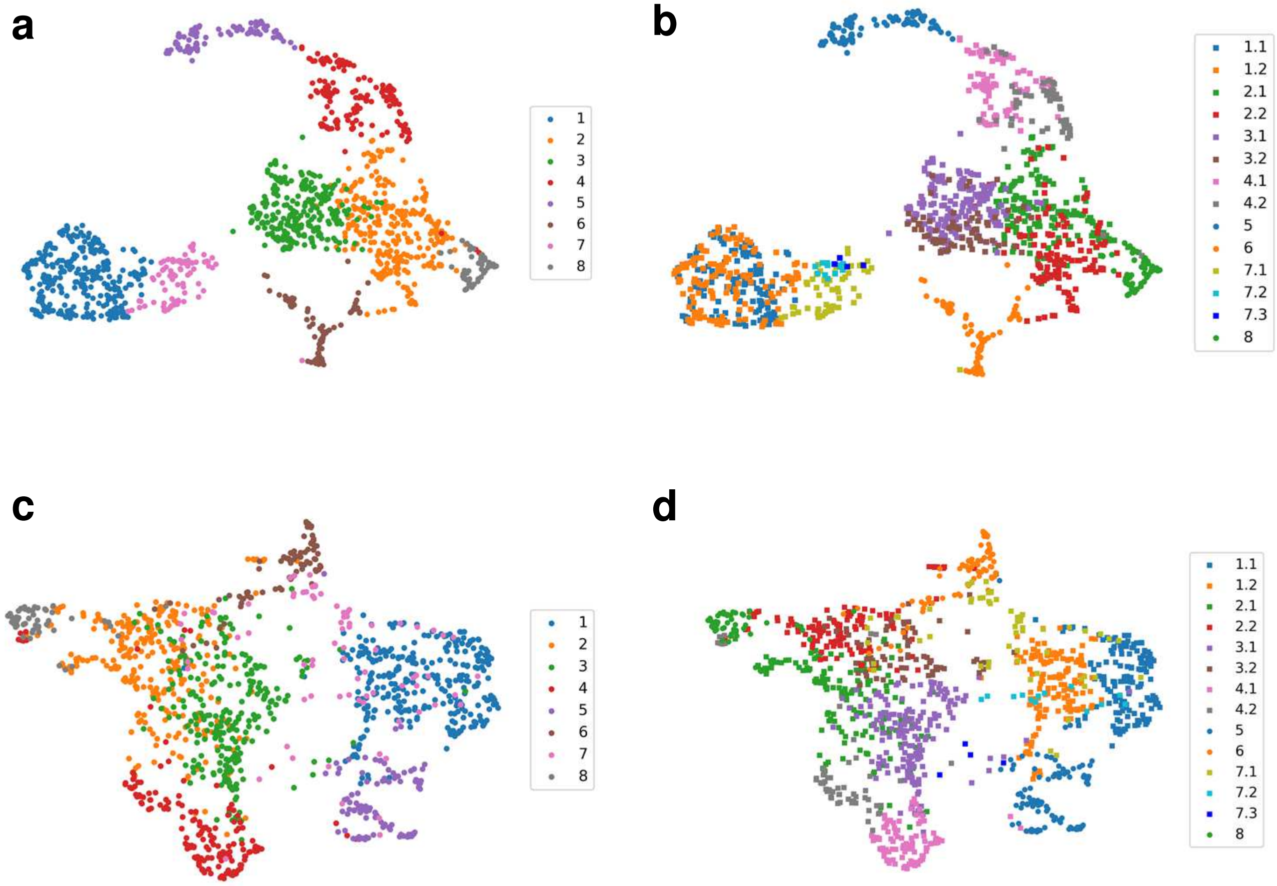

Supplementary Figure 6

Visualization of single-cell data of drosophila embryo.

(**a**) Visualization using UMAP embedding based on only scRNA-seq data labeled with clustering (PCA+Louvain) using scRNA-seq data. (**b**) Same embedding as (a) labeled with spatially localized subclusters (cell-cell distance based knn-graph+Louvain). (**c**, **d**) Similar to (a) and (b) with a UMAP embedding based on the spatial cell-cell distance.

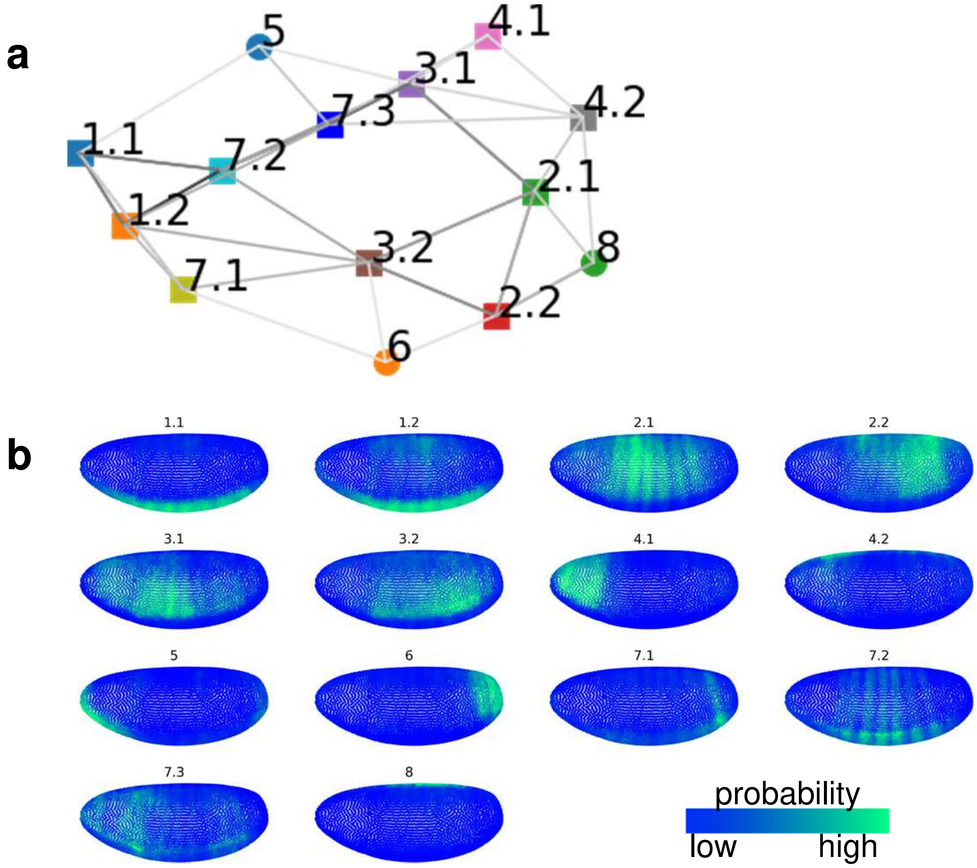

Supplementary Figure 7

Spatial summary of spatially localized subclusters in drosophila embryo data.

(**a**) A visualization of spatial arrangement of subclusters. The average cell-cell distance between cells from two subclusters are used to describe distances between the subclusters. This distance between subclusters is fed to umap to generate coordinates for visualization. In the network, each subcluster is connected to its three closest neighbors where a darker connection means a shorter distance. (**b**) Spatial origin distributions of the subclusters.

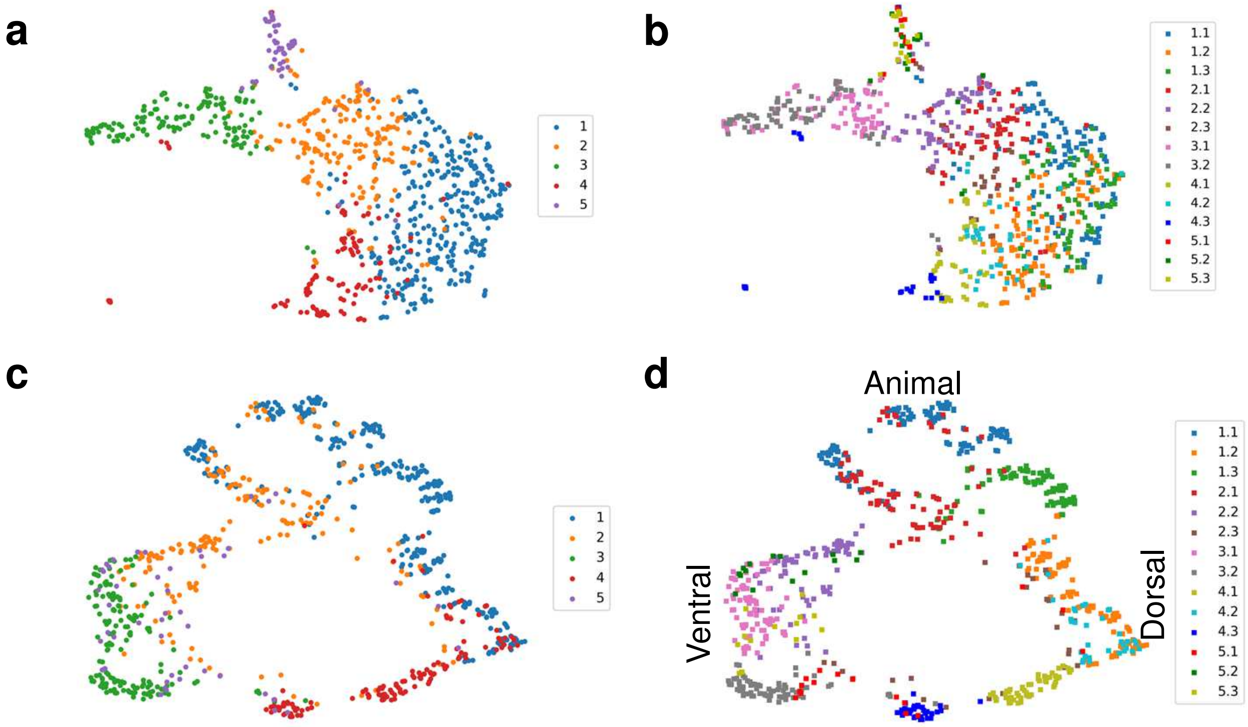

Supplementary Figure 8

Visualization of the first single-cell data of zebrafish embryo.

Both scRNA-seq data and spatial data are taken from (Satija, R. *et al., Nature Biotech.,* 2015). (**a**) Visualization using UMAP embedding based on only scRNA-seq data labeled with clustering (PCA+Louvain) using scRNA-seq data. (**b**) Same embedding as (a) labeled with spatially localized subclusters (cell-cell distance based knn-graph+Louvain). (**c**, **d**) Similar to (a) and (b) with a UMAP embedding based on the spatial cell-cell distance.

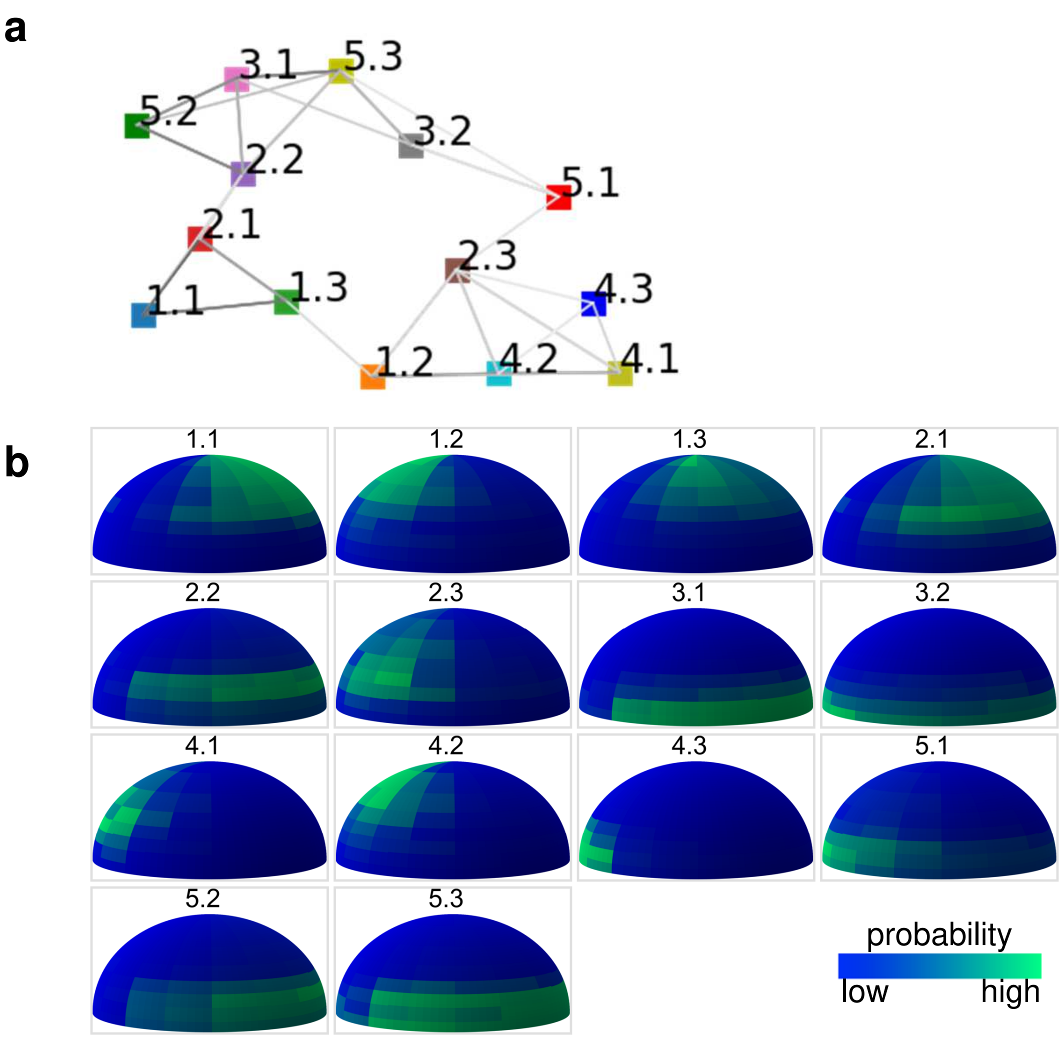

Supplementary Figure 9

Spatial summary of spatially localized subclusters in zebrafish embryo data.

Both scRNA-seq data and spatial data are taken from (Satija, R. *et al., Nature Biotech.,* 2015). (**a**) A visualization of spatial arrangement of subclusters. The average cell-cell distance between cells from two subclusters are used to describe distances between the subclusters. This distance between subclusters is fed to umap to generate coordinates for visualization. In the network, each subcluster is connected to its three closest neighbors where a darker connection means a smaller distance. (**b**) Spatial origin distributions of the subclusters.

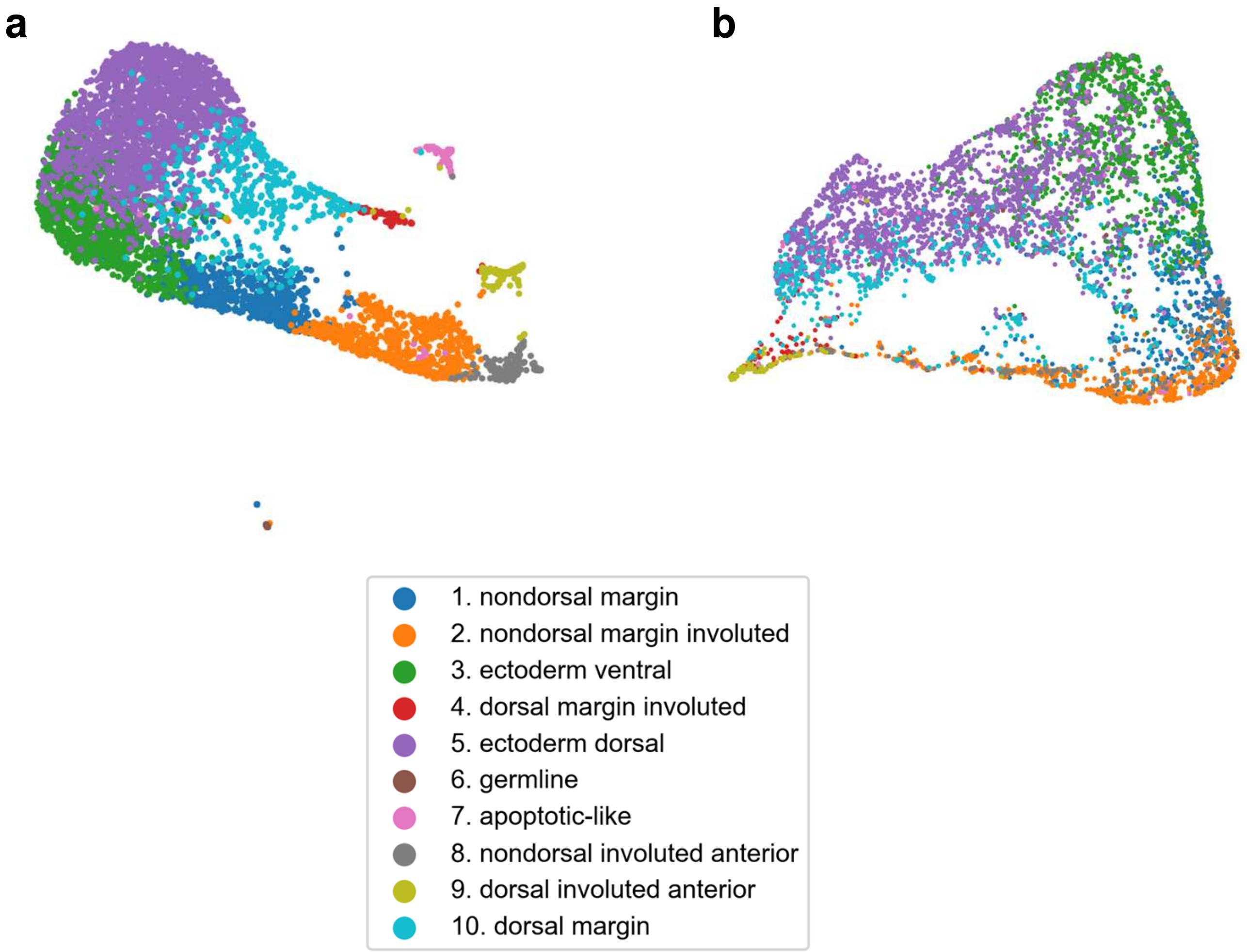

Supplementary Figure 10

Visualization of the second single-cell data of zebrafish embryo.

The spatial data is taken from (Satija, R. *et al., Nature Biotech.,* 2015) and the scRNA-seq data is from (Wagner, Daniel E. *et al.* Science, 2018). (**a**) Visualization using UMAP embedding based on only scRNA-seq data labeled with clustering from the original paper of the scRNA-seq data. (**b**) Similar to (a) with a UMAP embedding based on the spatial cell-cell distance.

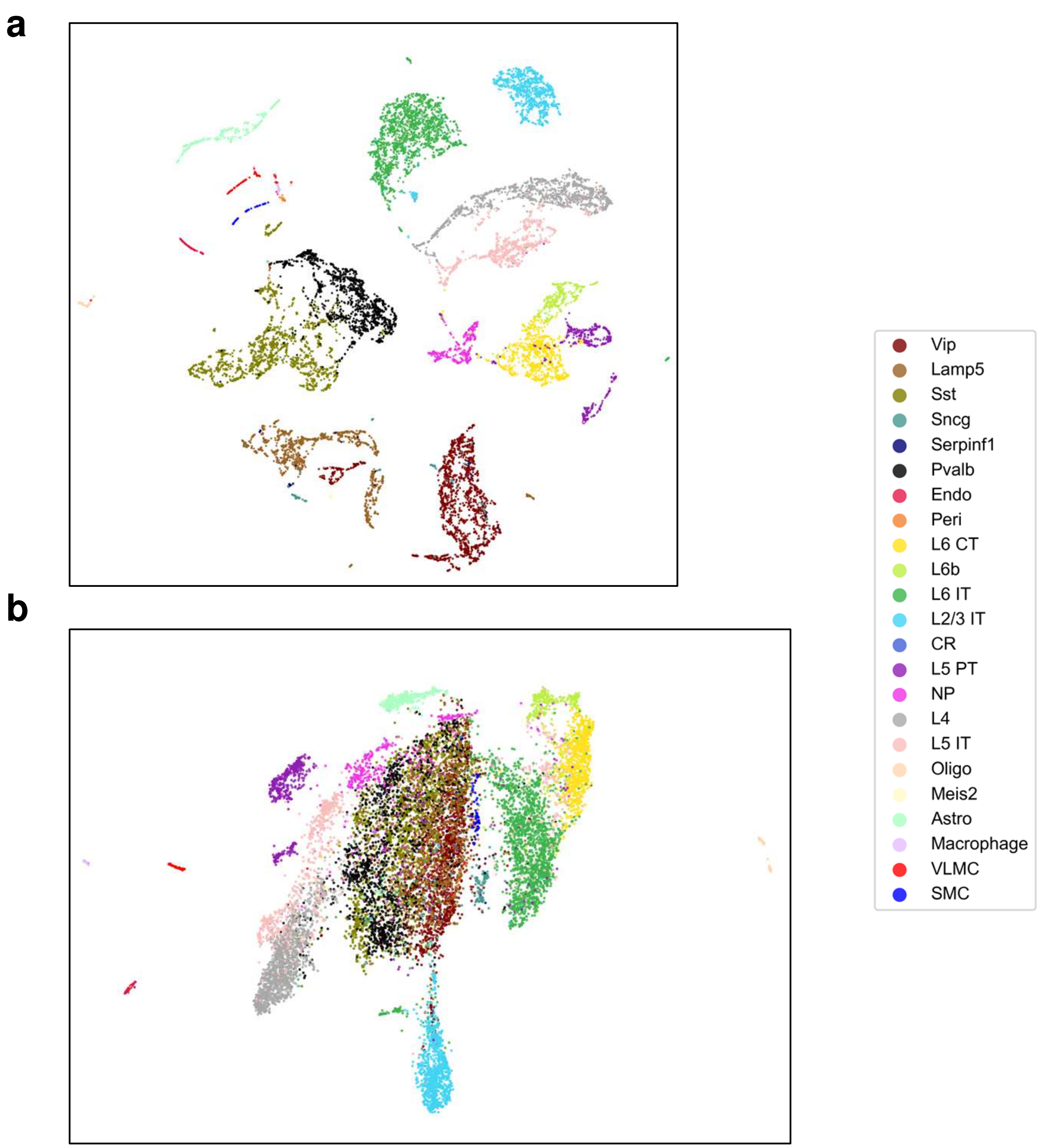

Supplementary Figure 11

Visualization of the mouse visual cortex scRNA-seq data.

(**a**) A visualization of the scRNA-seq data by applying UMAP following a PCA reduction with 20 components. (**b**) Similar to (a) but with UMAP embedding of the inferred spatial distance between cells.

a

b

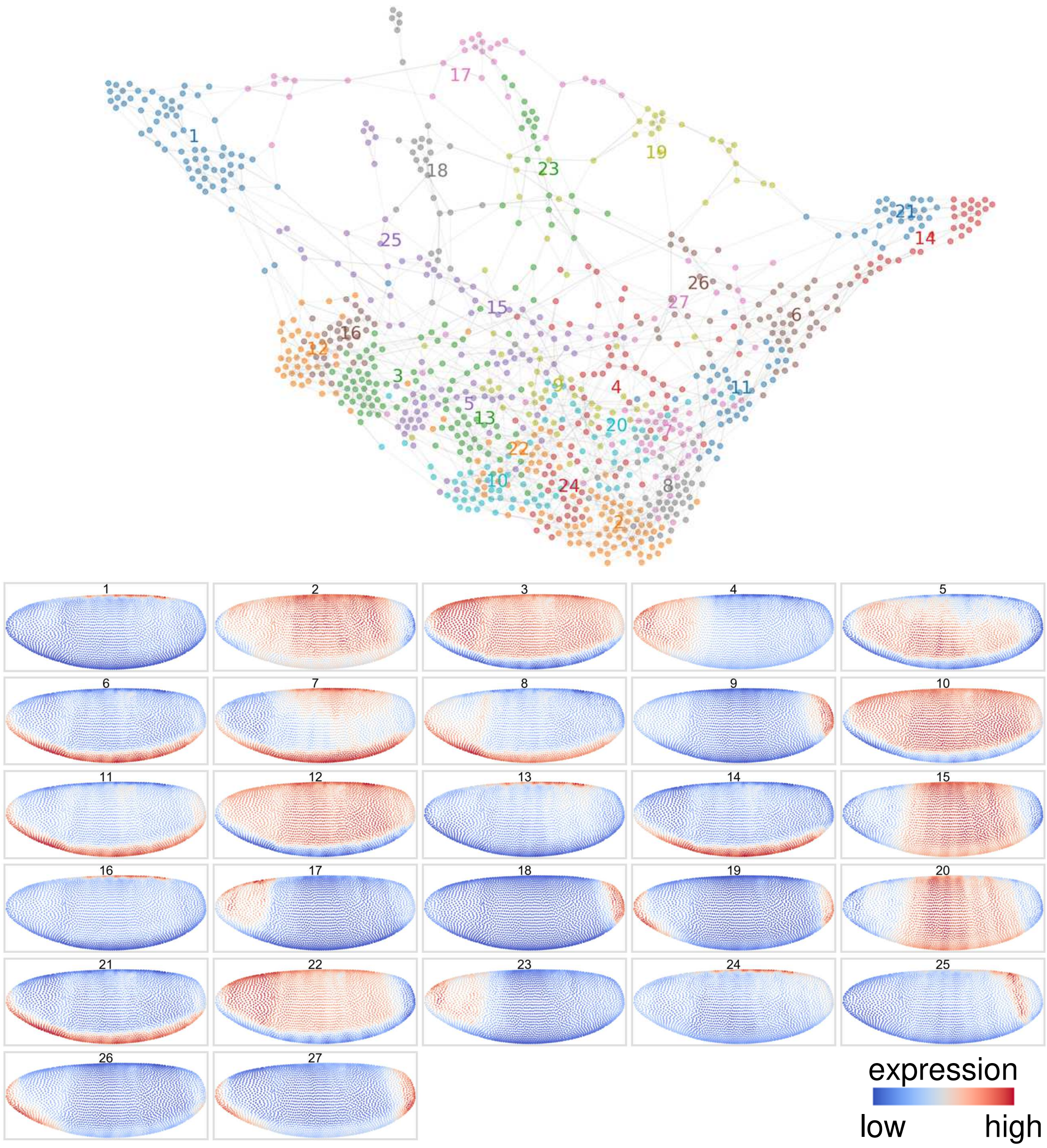

Supplementary Figure 12

Clustering of genes based on spatial pattern in drosophila embryo data.

(**a**) A visualization of k-nearest-neighbor graphs (k=3) of genes where each node represents a gene and an edge means the two genes have similar spatial expression pattern. The differences between genes are quantified using Wasserstein distance with the spatial distance between single cells as the cost matrix for the optimal transport problem. Only highly variable genes are considered. (**b**) Average spatial patterns mapped to the original geometry for the gene clusters.

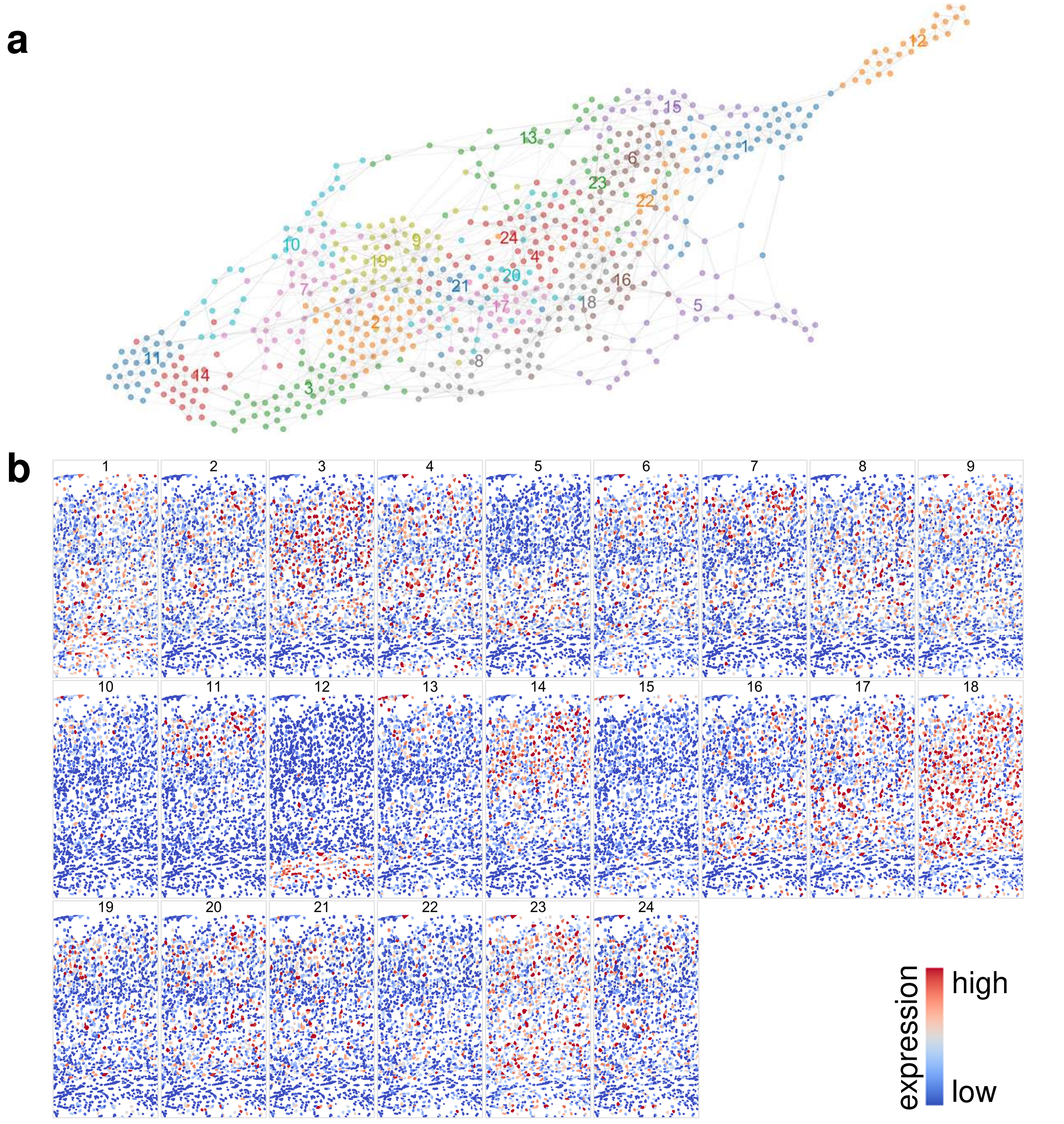

Supplementary Figure 13

Clustering of genes based on spatial pattern in mouse visual cortex data.

(**a**) A visualization of k-nearest-neighbor graphs (k=3) of genes where each node represents a gene and an edge means the two genes have similar spatial expression pattern. The differences between genes are quantified using Wasserstein distance computed using the spatial data. (**b**) Average expression patterns for the clusters.

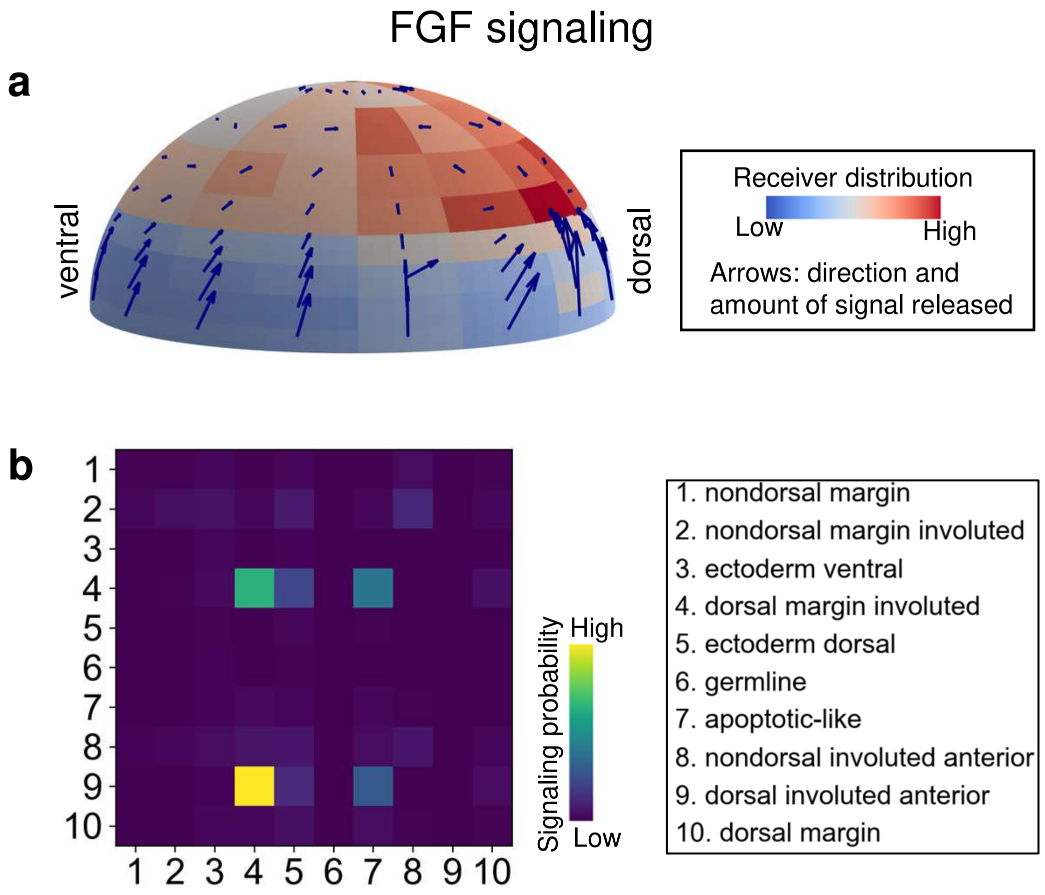

Supplementary Figure 14

Cell-cell communication in zebrafish embryo through FGF signaling inferred using optimal transport.

(**a**) (zebrafish embryo) FGF signaling interpolated from the SpaOTsc cell-cell communication matrix and mapped to space using the mapping between cells and positions. The length of arrow indicates the signal sending probability of the position and the color shows the estimated signal receiver probability distribution over space. (**b**) FGF signaling summarized into cell clusters.

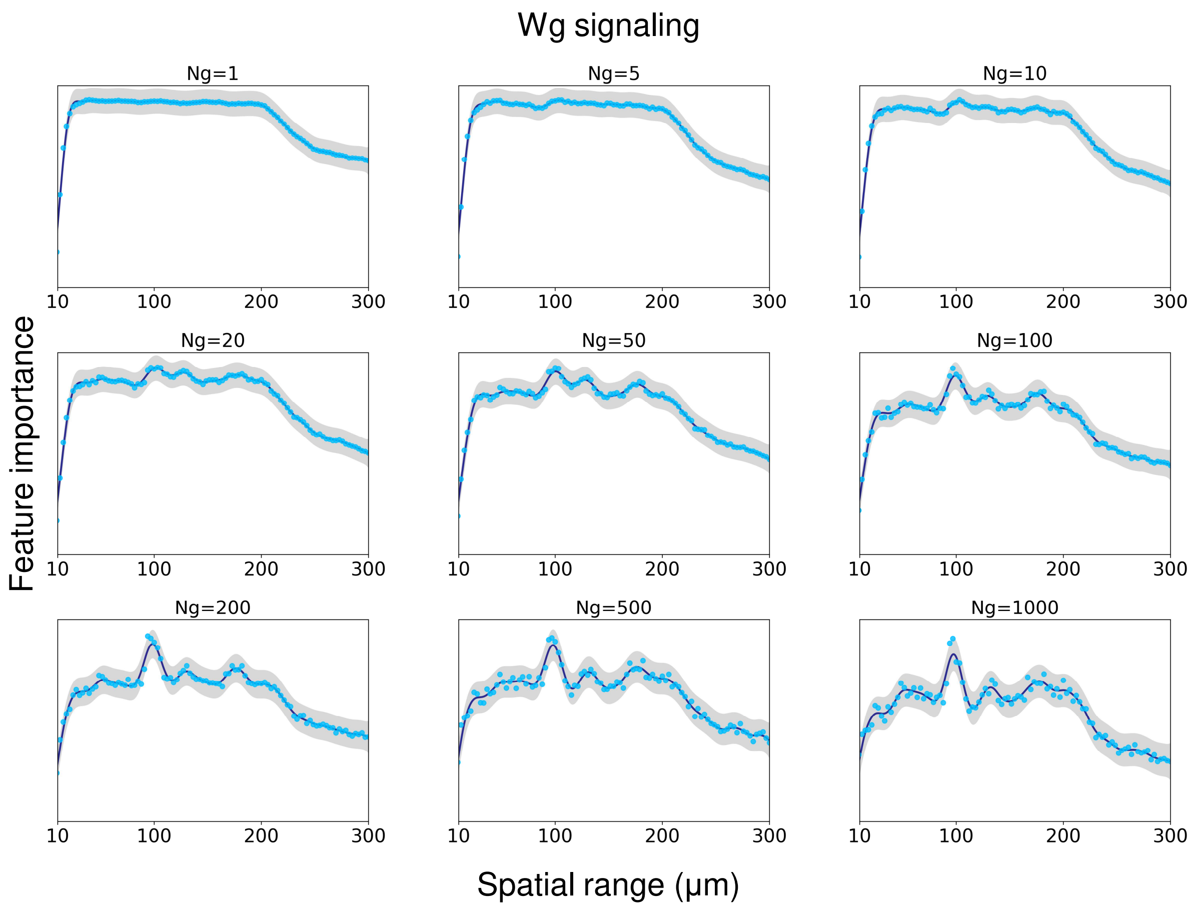

Supplementary Figure 15

Random forest inference for spatial range of Wg signaling with different number of background genes.

The inference of spatial range of Wg signaling in drosophila embryo data (ligand gene names: “wg”, “Wnt5” in data) using different number of background genes (Ng) in the random forest model. The grey band represents the 95% confidence interval. The experiments were repeated three times with similar results.

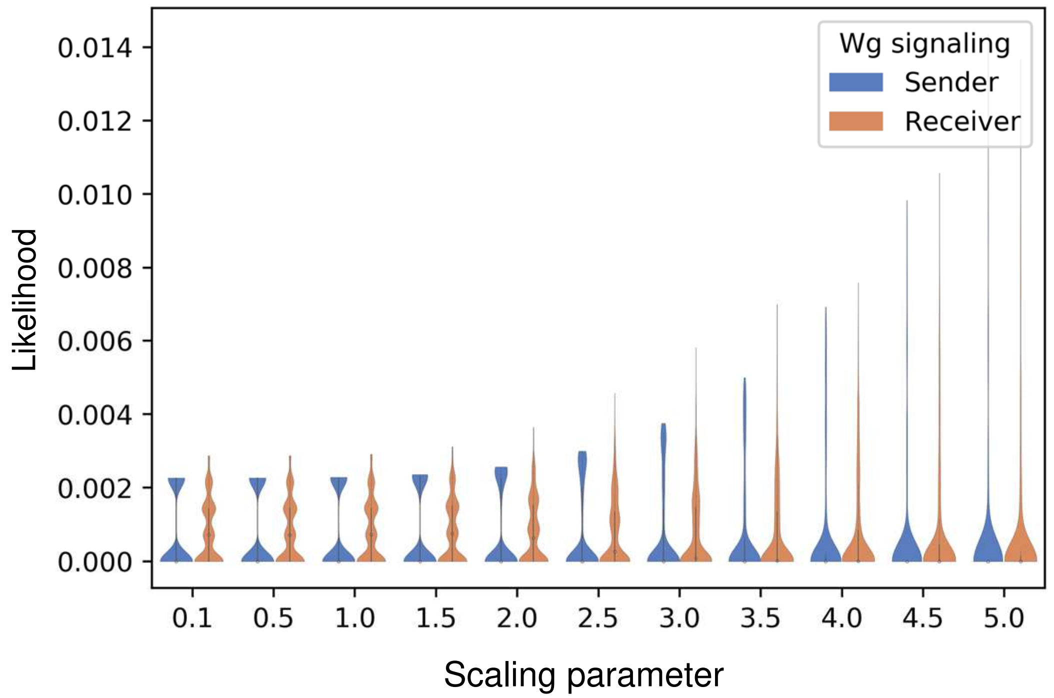

Supplementary Figure 16

The effect of choosing different scaling parameters determining the significance of gene expression.

The scaling parameter can be regarded as a soft cutoff for deciding whether a gene is significantly expressed when studying the cell-cell communications. It can be observed for this Wg signaling analysis of the drosophila embryo that more significant signal senders and receivers can be prioritized by increasing this scaling parameter. Inside the violin plots are standard boxplots (median, 25th perceltile, 75th percentile, the bigger of minimum value and 25th percentile – 1.5 interquartile range, and smaller of maximum value and 75th percentile + 1.5 interquartile range).

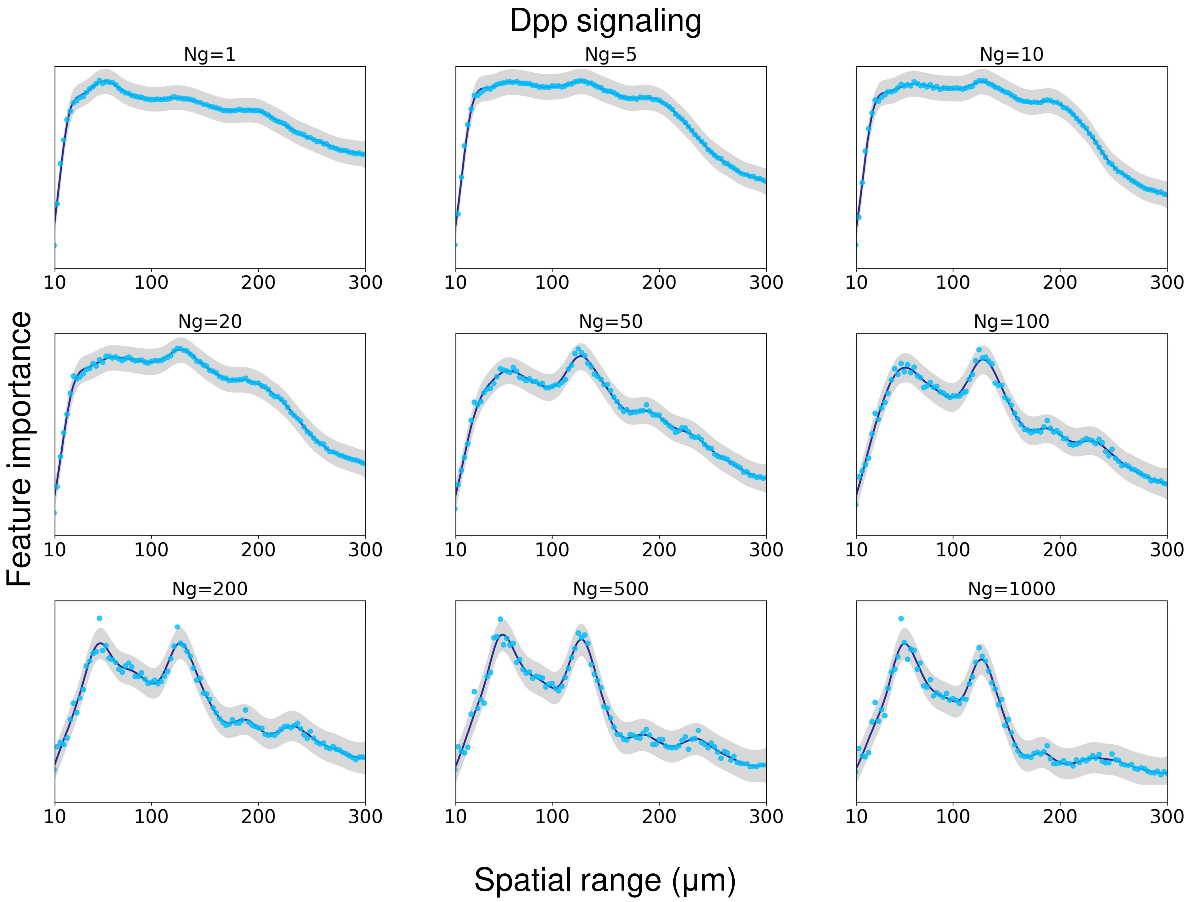

Supplementary Figure 17

Random forest inference for spatial range of Dpp signaling with different number of background genes.

The inference of spatial range of Dpp signaling in drosophila embryo data using different number of background genes (Ng) in the random forest model. The grey band represents the 95% confidence interval. The experiments were repeated three times with similar results.

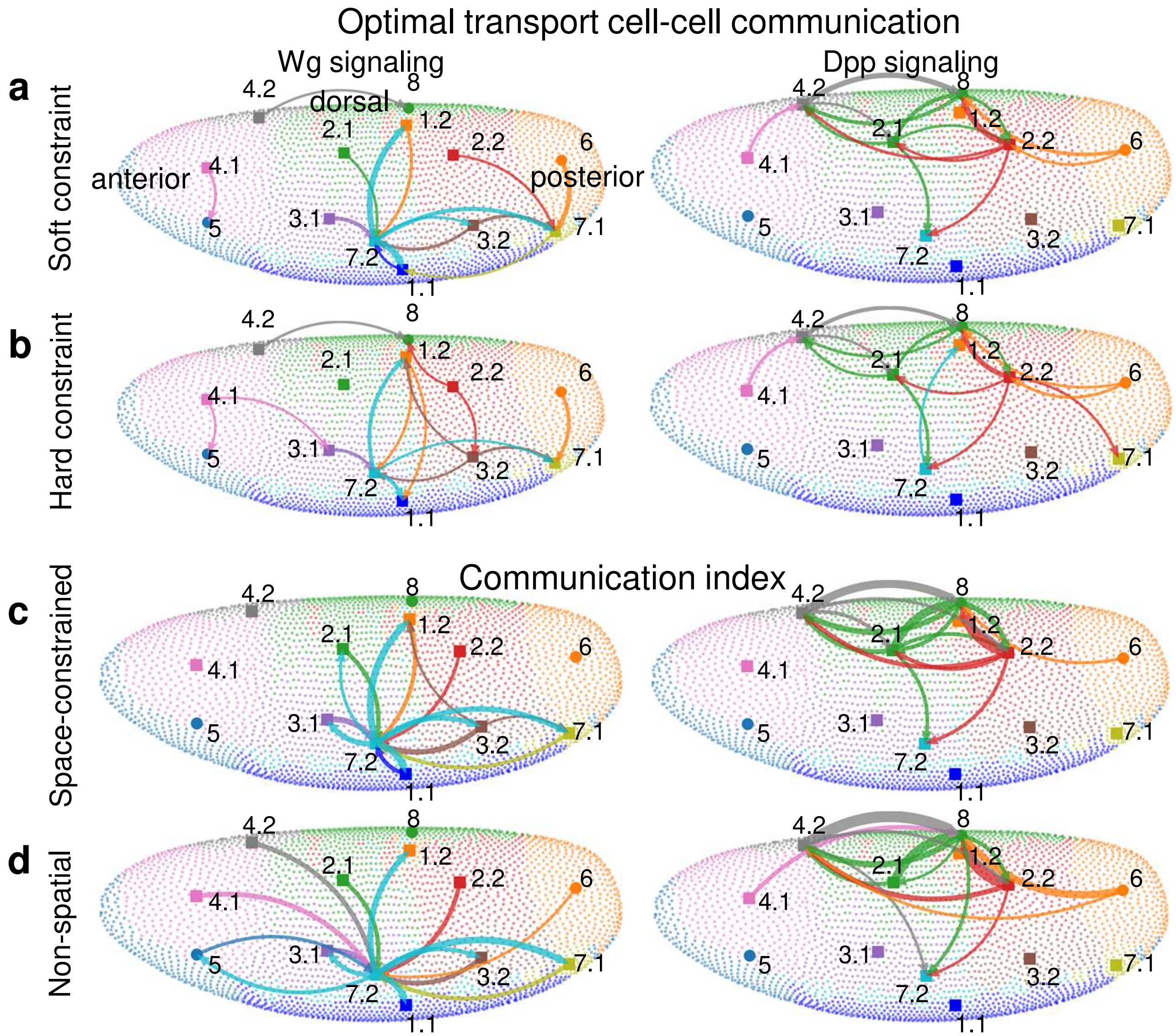

Supplementary Figure 18

Comparison of space-aware and –unaware cell-cell communication analysis at subcluster level.

The cell-cell communications are summarized to subcluster level. Top 15 communication arrows are plotted. The signaling ranges of Wg and Dpp are set to 100 μm and 125 μm respectively in (b) and (c) where explicit spatial constraints are used. The colors of points correspond to subcluster labels. (**a**) The cell-cell distance is used as cost matrix for the optimal transport inference but no distance cutoff is applied. (**b**) Similar to (a) with additional spatial distance cutoffs. (**c**) A communication index computed independently for each cell pair with a weight term addressing spatial constraint. (**d**) Similar to (c) without spatial information.

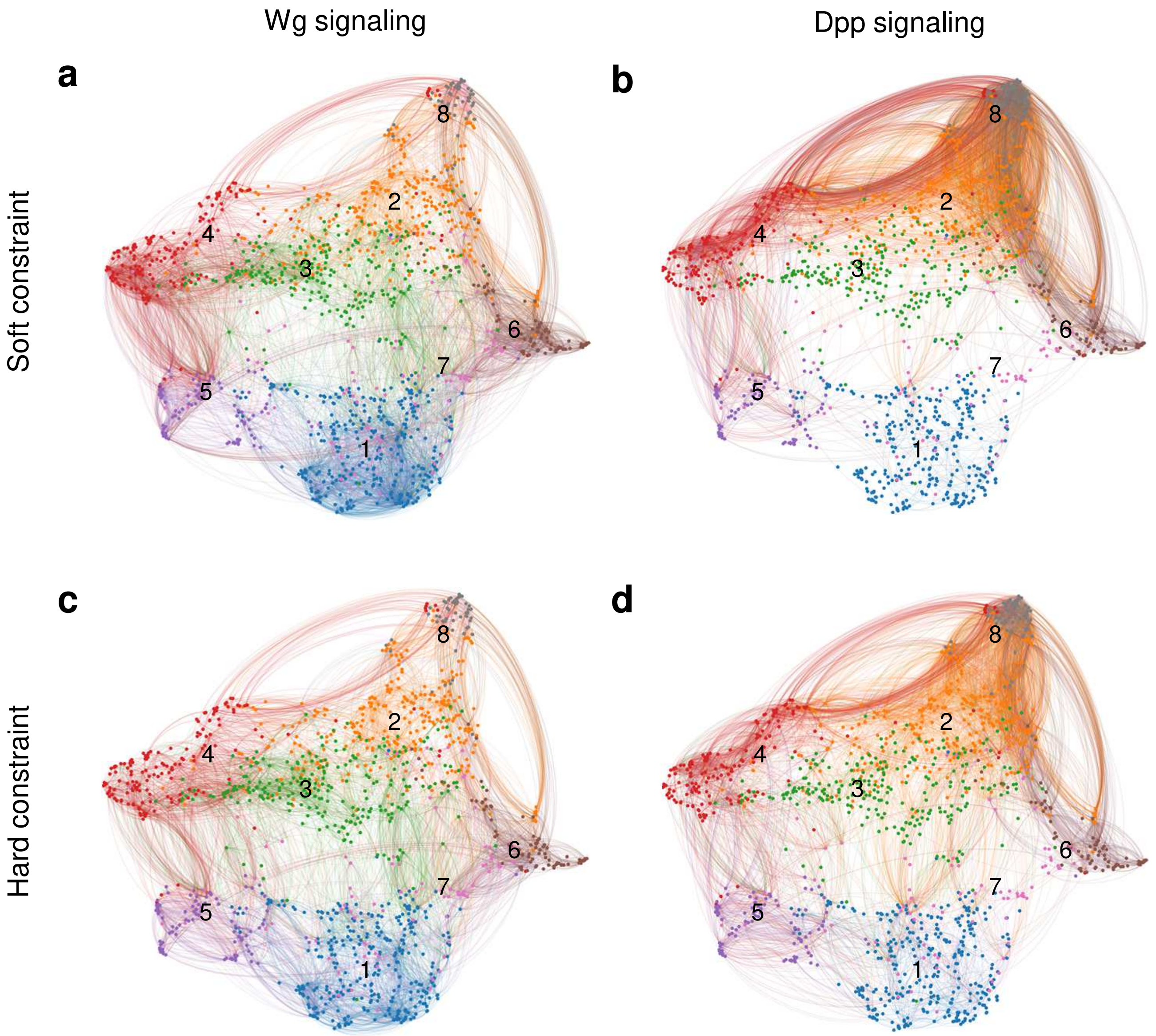

Supplementary Figure 19

Optimal transport communication with and without spatial distance cutoff.

The coordinates of cells in the 2d plots are obtained by UMAP map based on the inferred spatial distances between cells. The top 10000 cell-cell communications are plotted as curved lines. The color of the connections are same as the source cells. The colors of points correspond to subcluster labels. (**a, b**) Spatial cell-cell distance is used as transport cost matrix and no cutoff distance for signaling is used. (**c**, **d**) Similar to (a) and (b) with additional explicit spatial distance cutoffs (100 μm for Wg and 125 μm for Dpp).

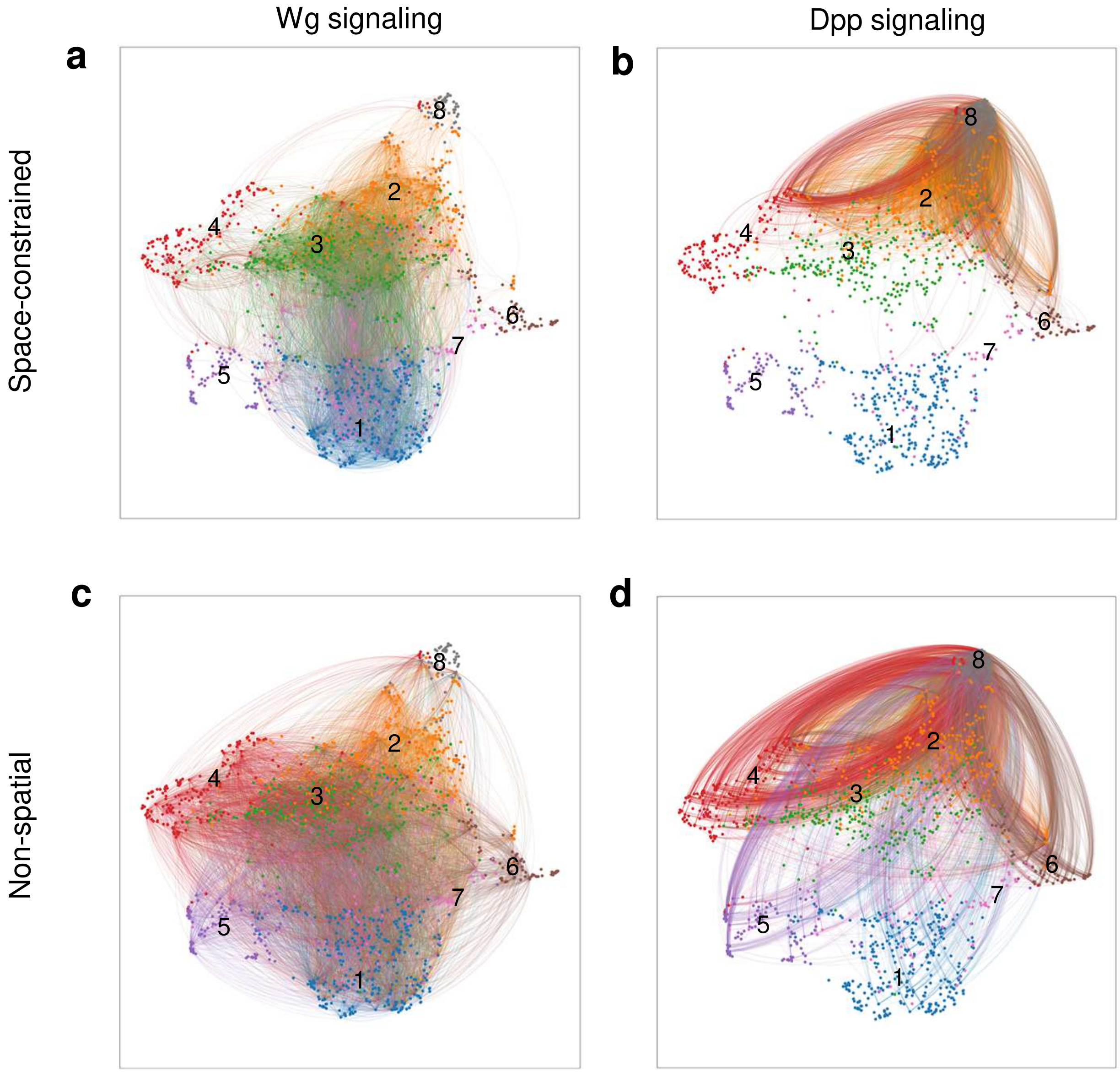

Supplementary Figure 20

Communication index with and without spatial constraint.

The coordinates of cells in the 2d plots are obtained by UMAP map based on the inferred spatial distances between cells. The top 10000 cell-cell communications are plotted as curved lines. The color of the connections are same as the source cells. The colors of points correspond to subcluster labels. (**a**) Space-constrained analysis of Wg signaling under a spatial distance of 100 μm. (**b**) Space-constrained analysis of Dpp signaling under a spatial distance of 120 μm. (**c**, **d**) Top communications identified without spatial information.

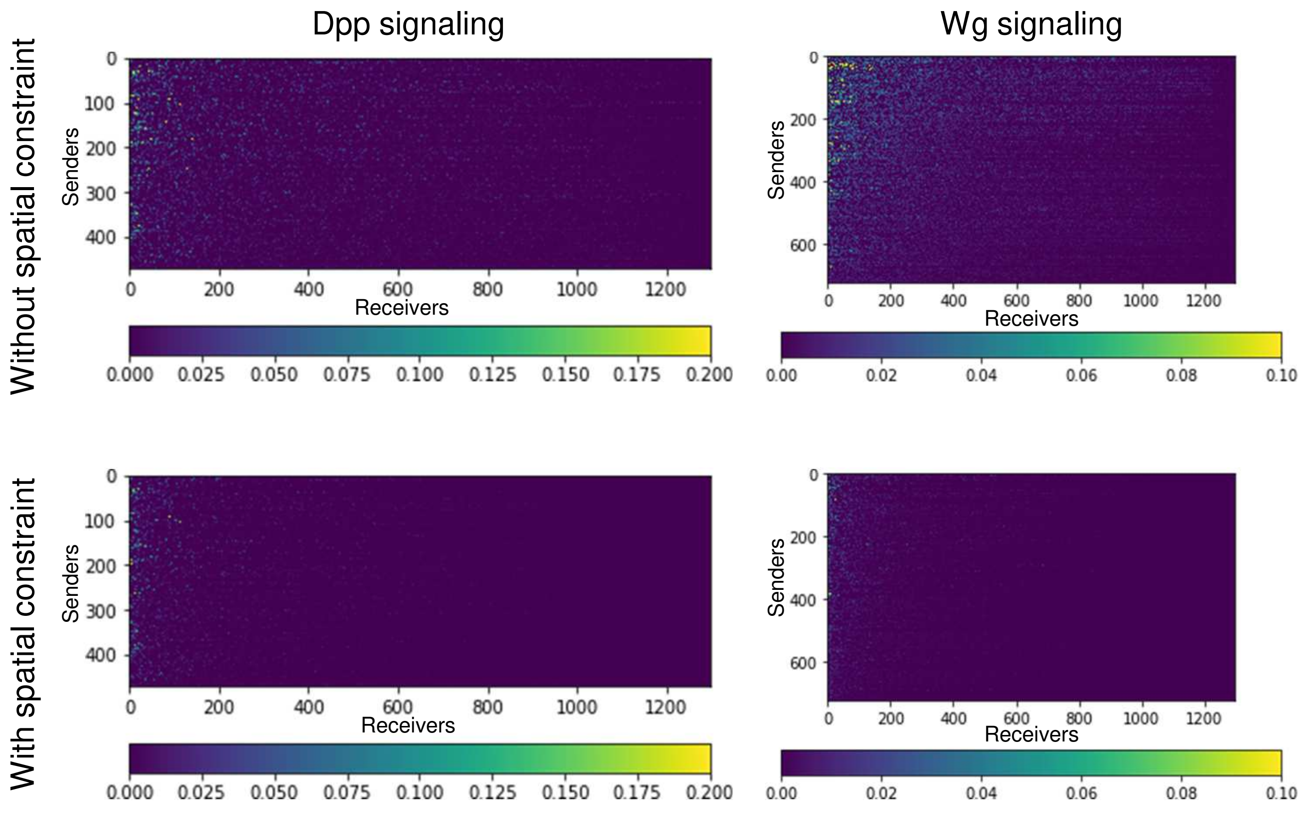

Supplementary Figure 21

The effect of adding spatial constraint to optimal transport cell-cell communication inference in drosophila embryo.

In the heat map each row corresponds to a signal sender cell and the signal receivers in each row is sorted based on their distance to the sender cell corresponding to that row.

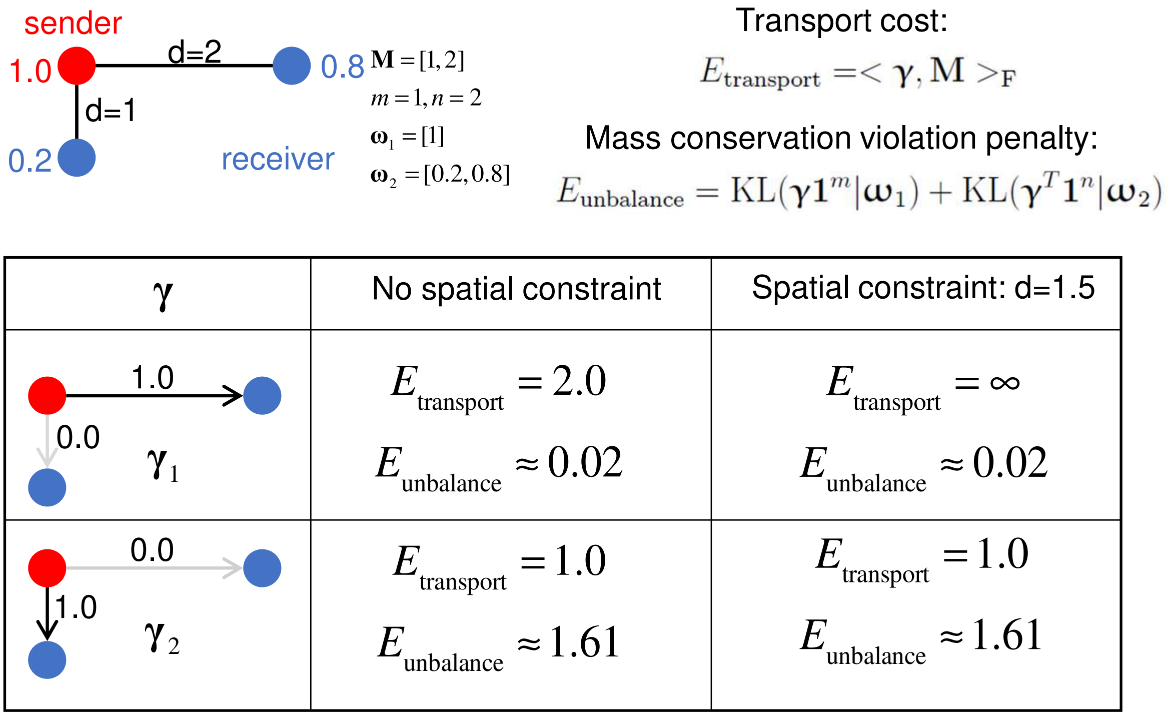

Supplementary Figure 22

An example showing the effect of adding spatial constraint to optimal transport cell-cell communication inference.

We consider an example system with one signal sending cell and two signal receiving cells. In the two example transport maps: one that transports all mass from the signal sender to a strong but distant signal receiver and one that transports all mass from the signal sender to a nearby but moderate signal receiver. When a spatial constraint is applied, the second transport map becomes more favorable which had higher cost than the first one when there is no spatial constrain.

EGF/EGFR signaling

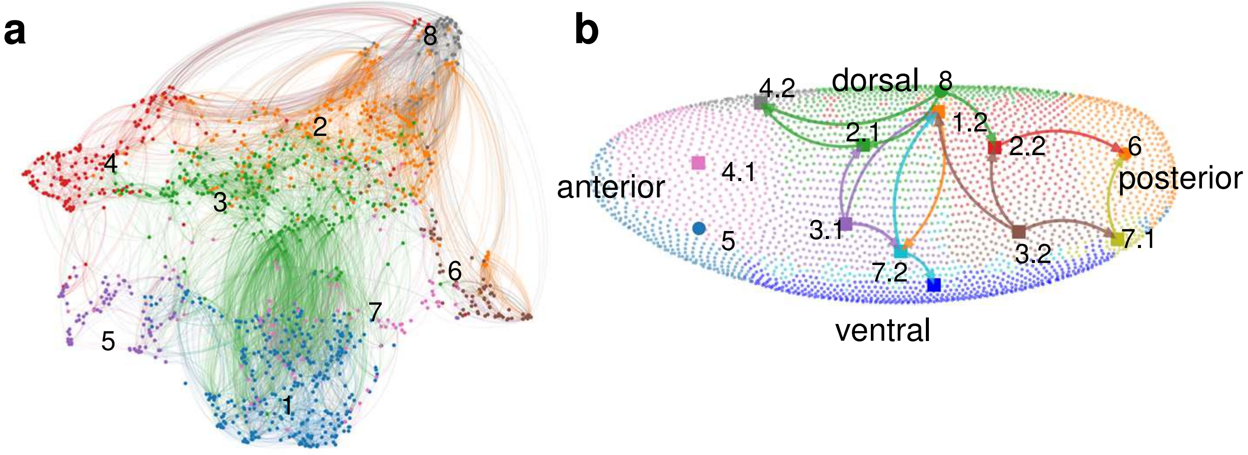

Supplementary Figure 23

Cell-cell communication in drosophila embryo through EGF/EGFR signaling inferred using optimal transport.

(**a**) The coordinates of cells in the 2d plots are obtained by UMAP map based on the inferred spatial distances between cells. The top 10000 cell-cell communications are plotted as curved lines. The color of the connections are same as the source cells. The colors of points correspond to cluster labels. (**b**) The communications summarized to subclusters.

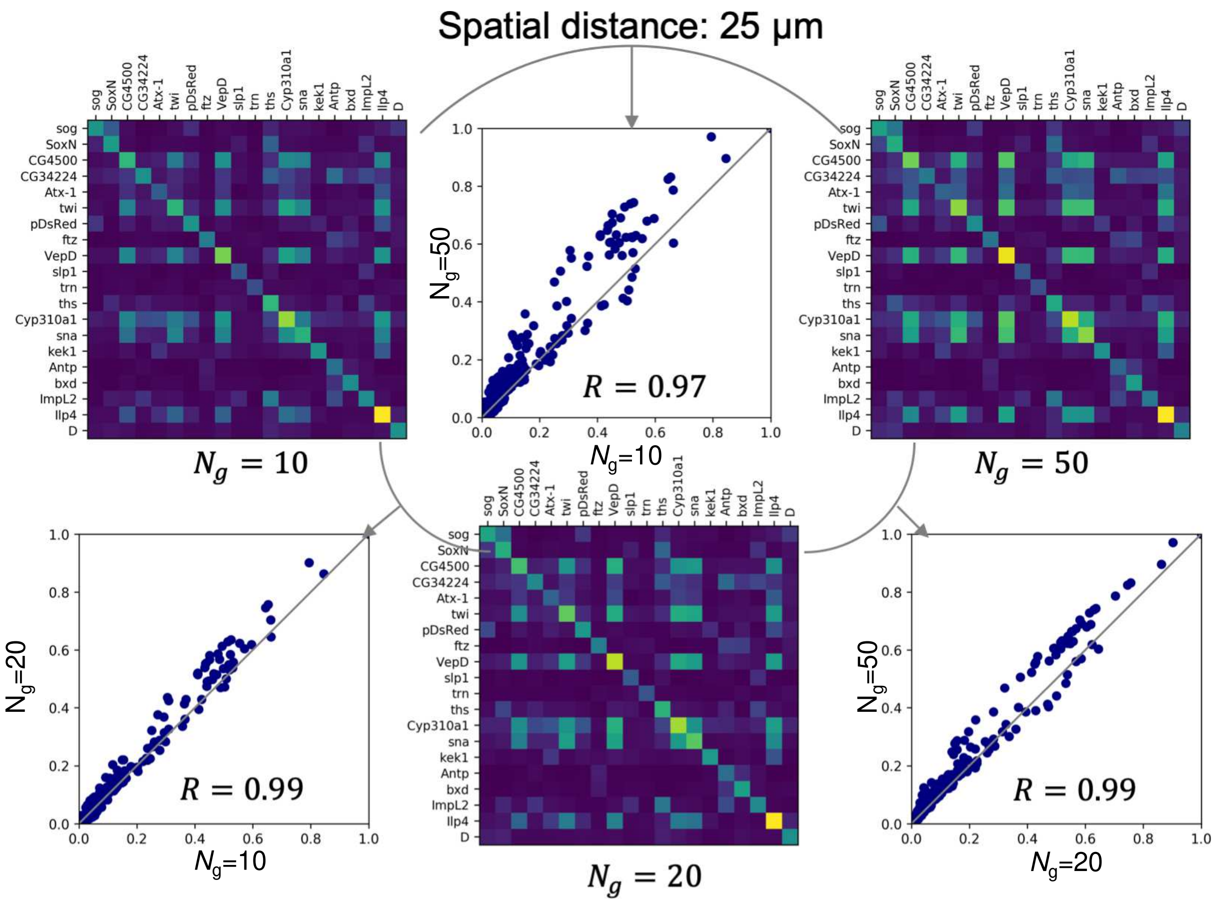

Supplementary Figure 24

PID inference of intercellular gene regulation in drosophila data with different number of background genes under a spatial range of 25 μm.

The ith row and jth column quantifies the unique information provided about the jth gene (target gene) by the ith gene (source gene) in a spatial neighborhood of 25 μm. Different numbers of background genes (N_g=10, 20, 50) are considered when computing the unique information. The genes with the highest intracellular correlation with the target genes are selected as background genes respectively for each target gene. The scatter plot shows comparison of the nonzero entries of the matrices quantified by the Pearson correlation coefficient.

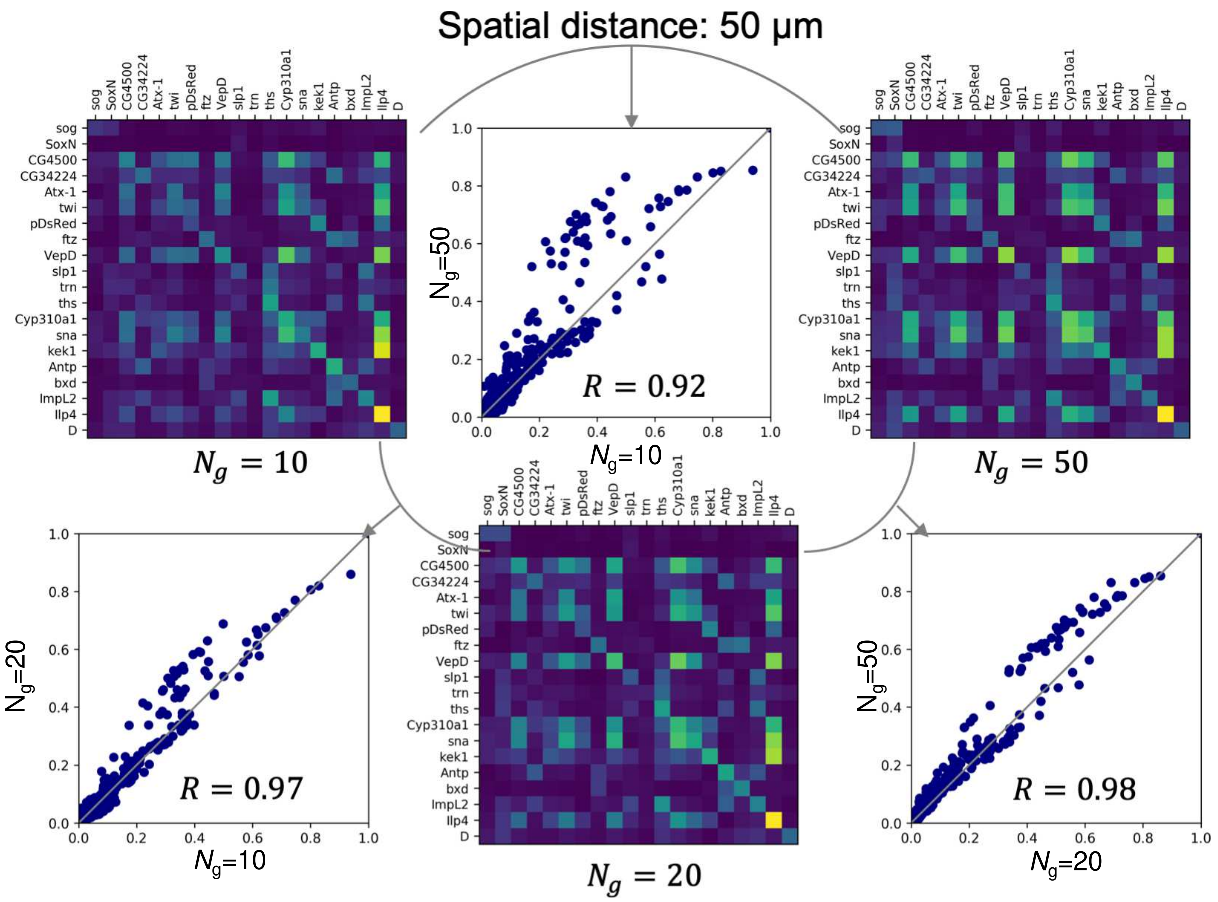

Supplementary Figure 25

PID inference of intercellular gene regulation in drosophila data with different number of background genes under a spatial range of 50 μm.

The ith row and jth column quantifies the unique information provided about the jth gene (target gene) by the ith gene (source gene) in a spatial neighborhood of 50 μm. Different numbers of background genes (N_g=10, 20, 50) are considered when computing the unique information. The genes with the highest intracellular correlation with the target genes are selected as background genes respectively for each target gene. The scatter plot shows comparison of the nonzero entries of the matrices quantified by the Pearson correlation coefficient.

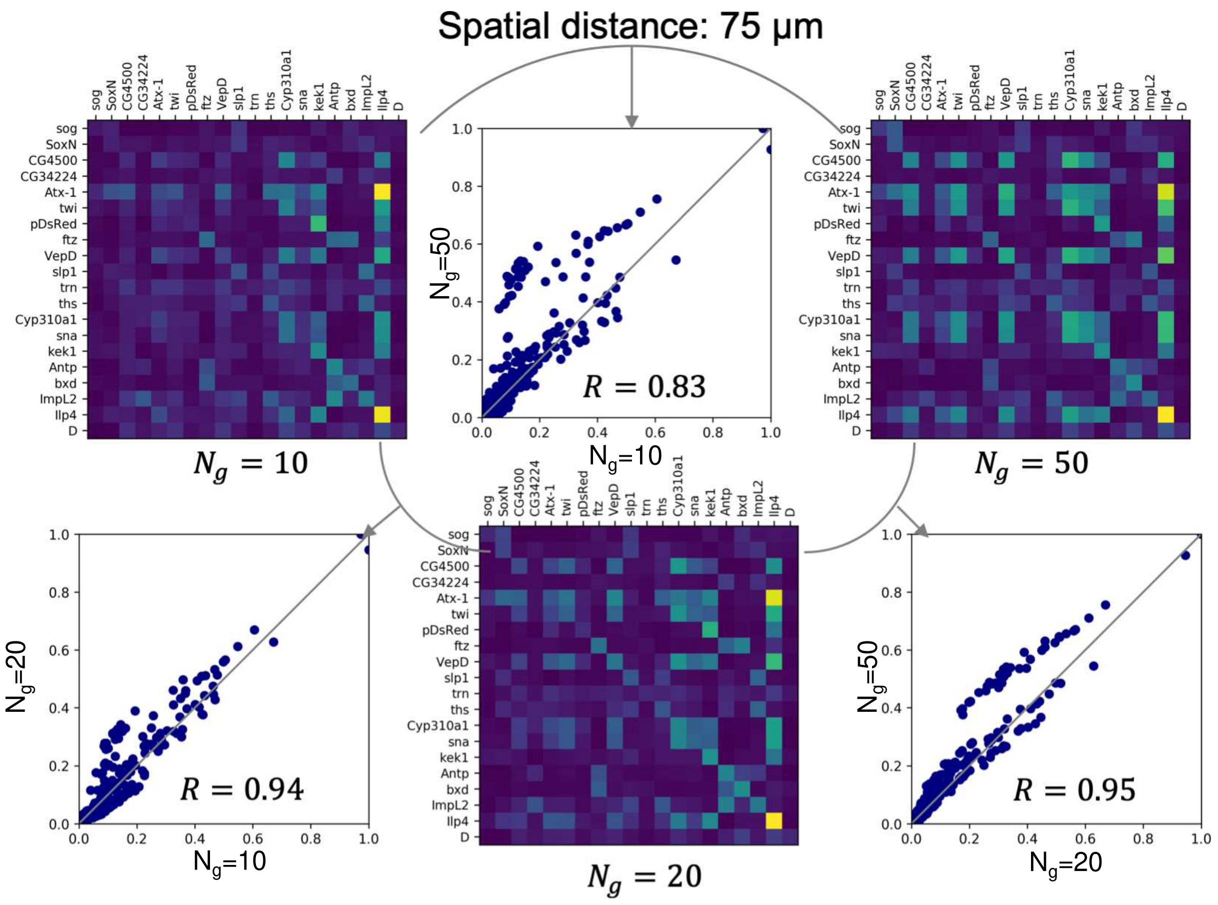

Supplementary Figure 26

PID inference of intercellular gene regulation in drosophila data with different number of background genes under a spatial range of 75 μm.

The ith row and jth column quantifies the unique information provided about the jth gene (target gene) by the ith gene (source gene) in a spatial neighborhood of 75 μm. Different numbers of background genes (N_g=10, 20, 50) are considered when computing the unique information. The genes with the highest intracellular correlation with the target genes are selected as background genes respectively for each target gene. The scatter plot shows comparison of the nonzero entries of the matrices quantified by the Pearson correlation coefficient.

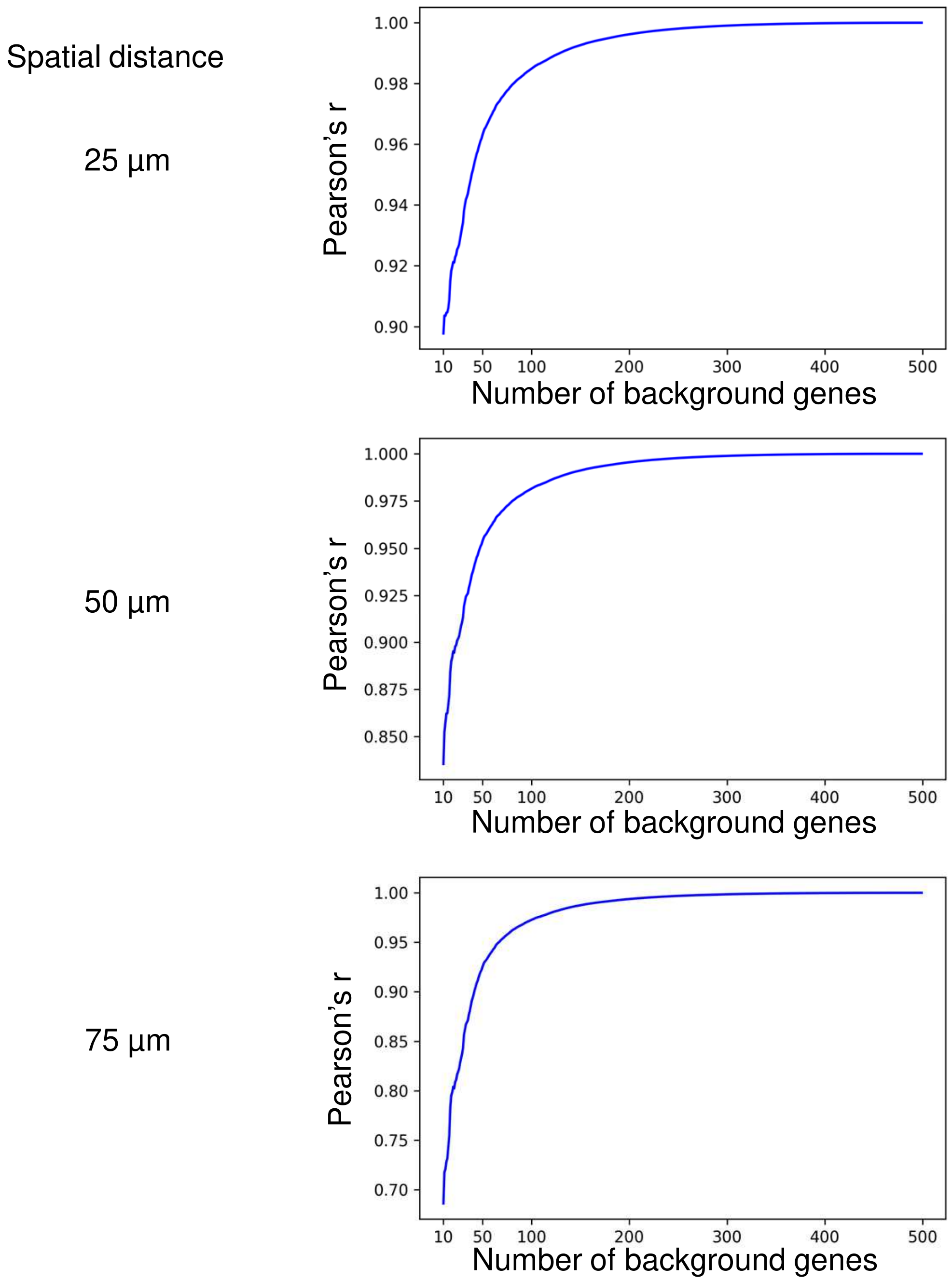

Supplementary Figure 27

PID inference of intercellular gene regulation in drosophila data with different number of background genes compared to using 500 background genes.

The gene-gene information flow among the 20 most variable genes in the drosophila datasets were inferred using various numbers of background genes ranging from 10 to 500. All obtained gene-gene interactions were compared to using 500 background genes using the Pearson's correlation coefficients.

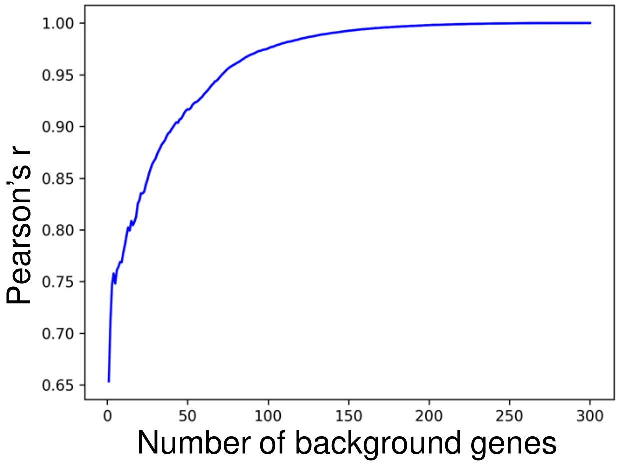

Supplementary Figure 28

PID inference of intercellular gene regulation in the mouse visual cortex data for with different number of background genes compared to using 300 background genes.

The gene-gene information flow among the 20 most variable genes in the drosophila datasets were inferred using various numbers of background genes ranging from 1 to 300. All obtained gene-gene interactions were compared to using 300 background genes using the Pearson's correlation coefficients. The distance scale is set to 1/10 of the longest spatial distance among the single cells.

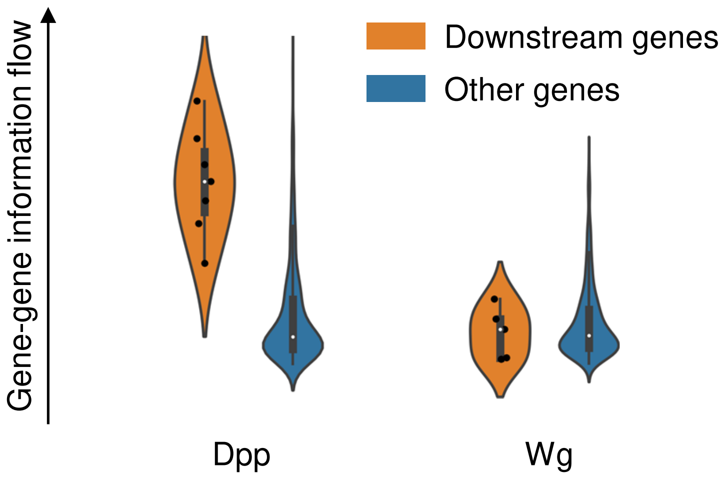

Supplementary Figure 29

Gene-gene information flows for signaling pathways.

We evaluate the PID approach to quantifying gene-gene information flow by applying it to known ligands and downstream genes involved in intercellular signaling. To this end, we compute the spatial gene-gene information flow from the ligand genes to a collection of highly variable genes. The flows to downstream target genes in the signaling pathway and to other genes are compared. The former is expected to be higher than the later. Spatial ranges of 125 μm and 100 μm (inferred by SpaOTsc) are used for Dpp signaling and Wg signaling respectively. Inside the violin plots are boxplots (median, 25th perceltile, 75th percentile, the bigger of minimum value and 25th percentile – 1.5 interquartile range, and smaller of maximum value and 75th percentile + 1.5 interquartile range). The numbers of data points for the violin plots from left to right are 7, 941, 5, 943.

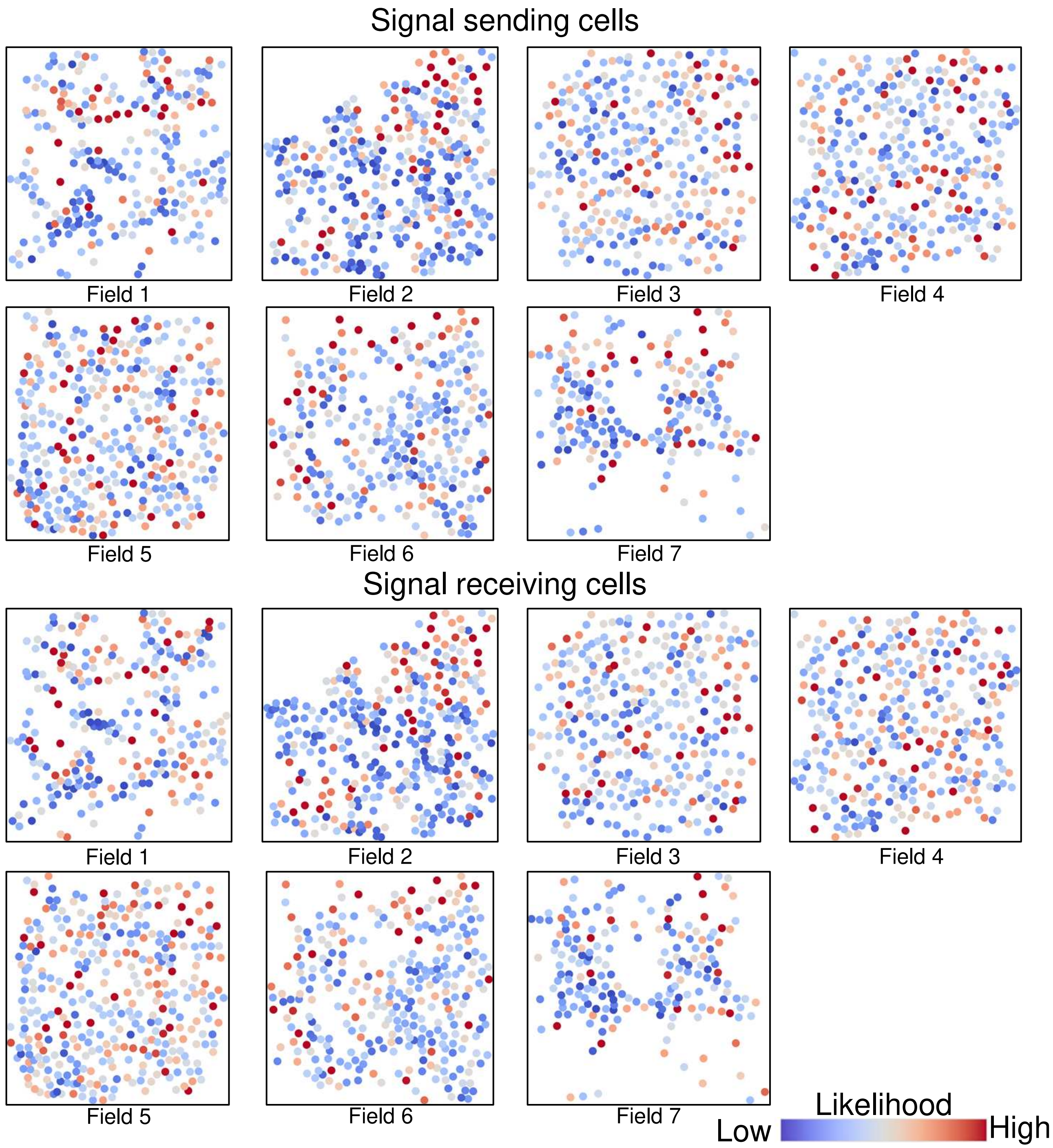

Supplementary Figure 30

The likelihood of signal sending and receiving cells of the RNA seqFISH+ data of mouse olfactory bulb.

The seven fields in the RNA seqFISH+ data are plotted separately.

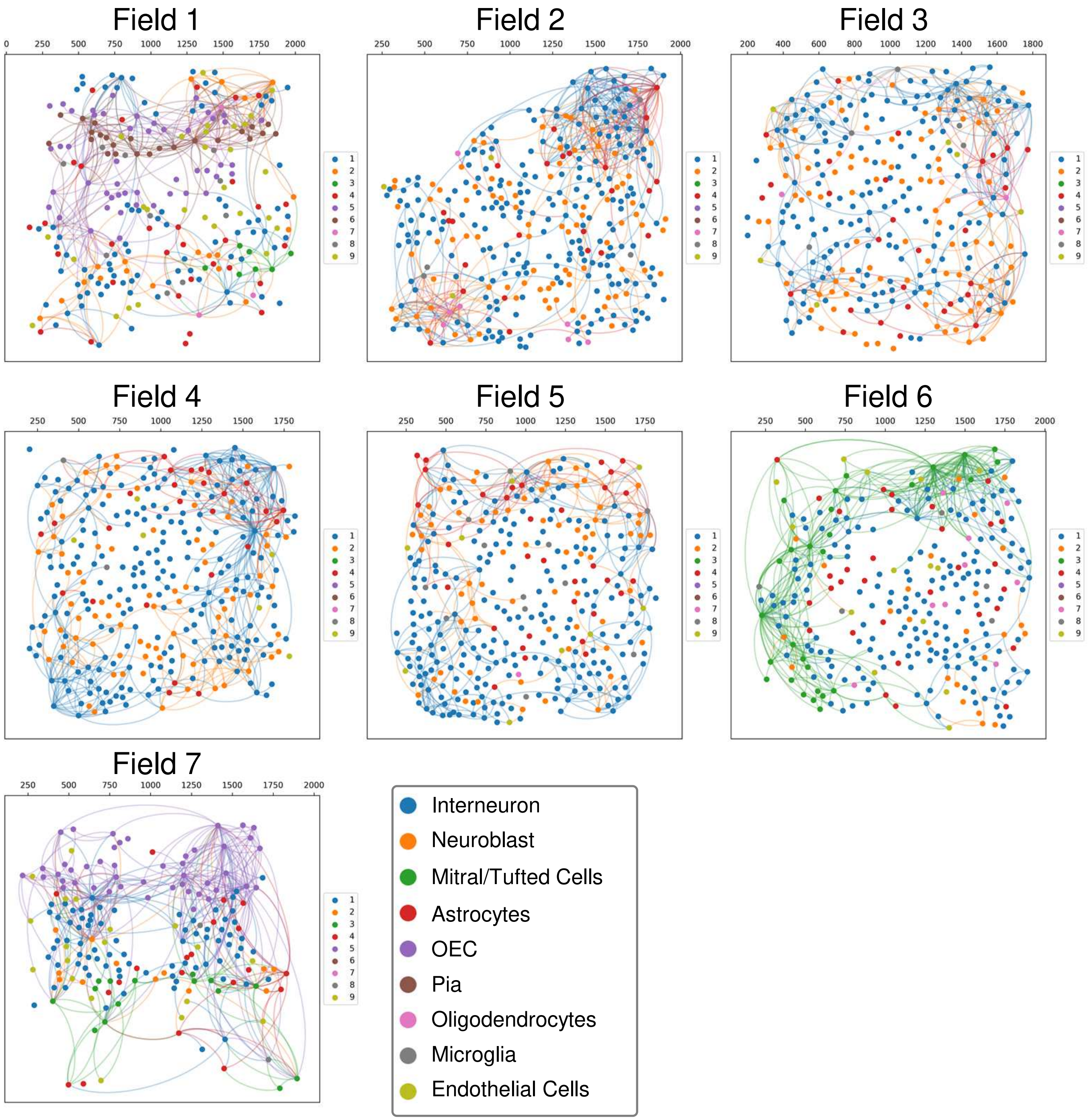

Supplementary Figure 31

Inferred cell-cell communications in the RNA seqFISH+ data of mouse olfactory bulb.

The color of links correspond to the signal sending cells. The cell-cell communications were inferred for each field separately.

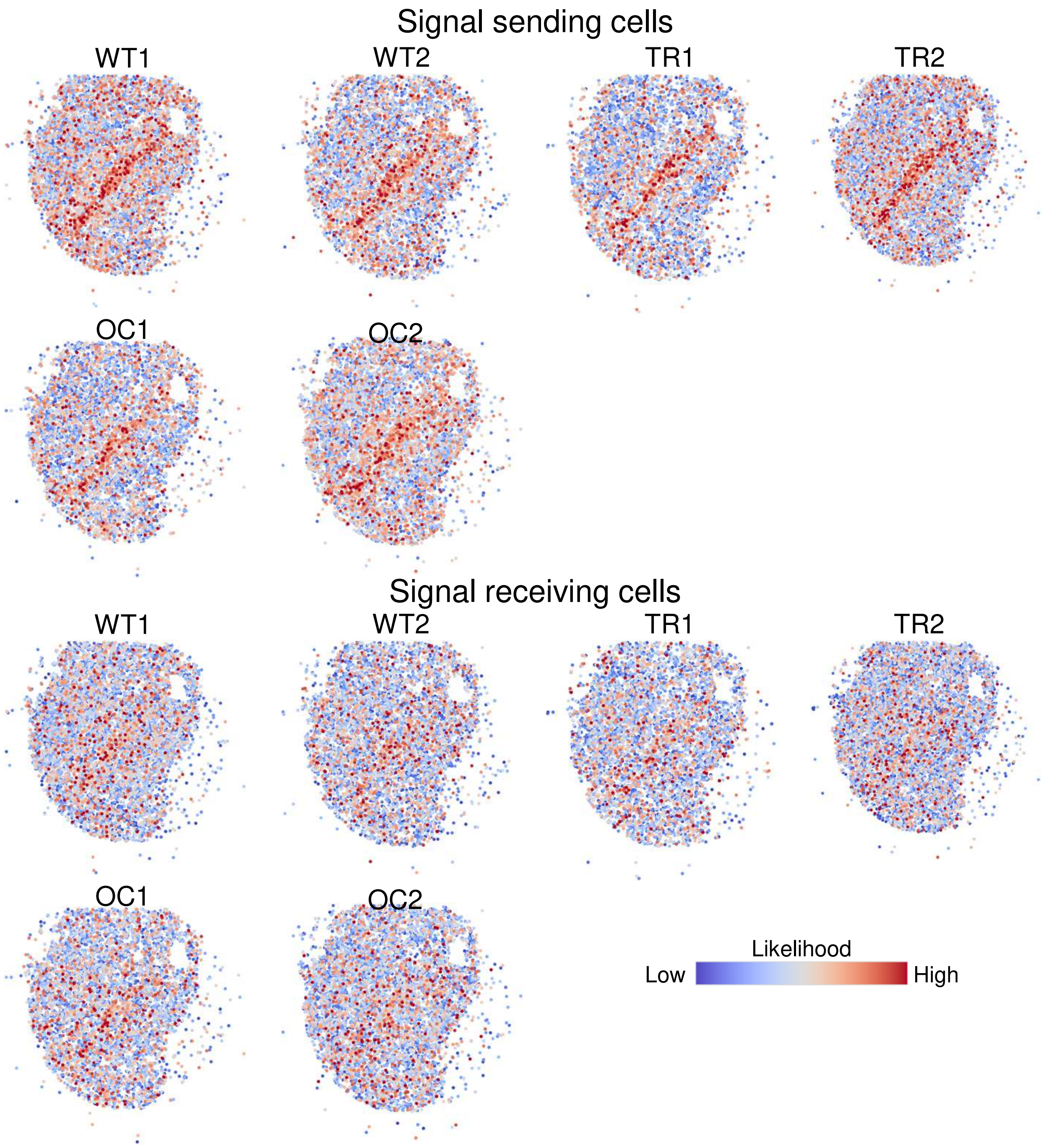

Supplementary Figure 32

The likelihood of signal sending and receiving cells of the scRNA-seq data of mouse olfactory bulb mapped to the space of Slide-seq data.

Each dot corresponds to a single cell in the scRNA-seq data and the position is determined based on the SpaOTsc mapping of this scRNA-seq data and the reference Slide-seq data.

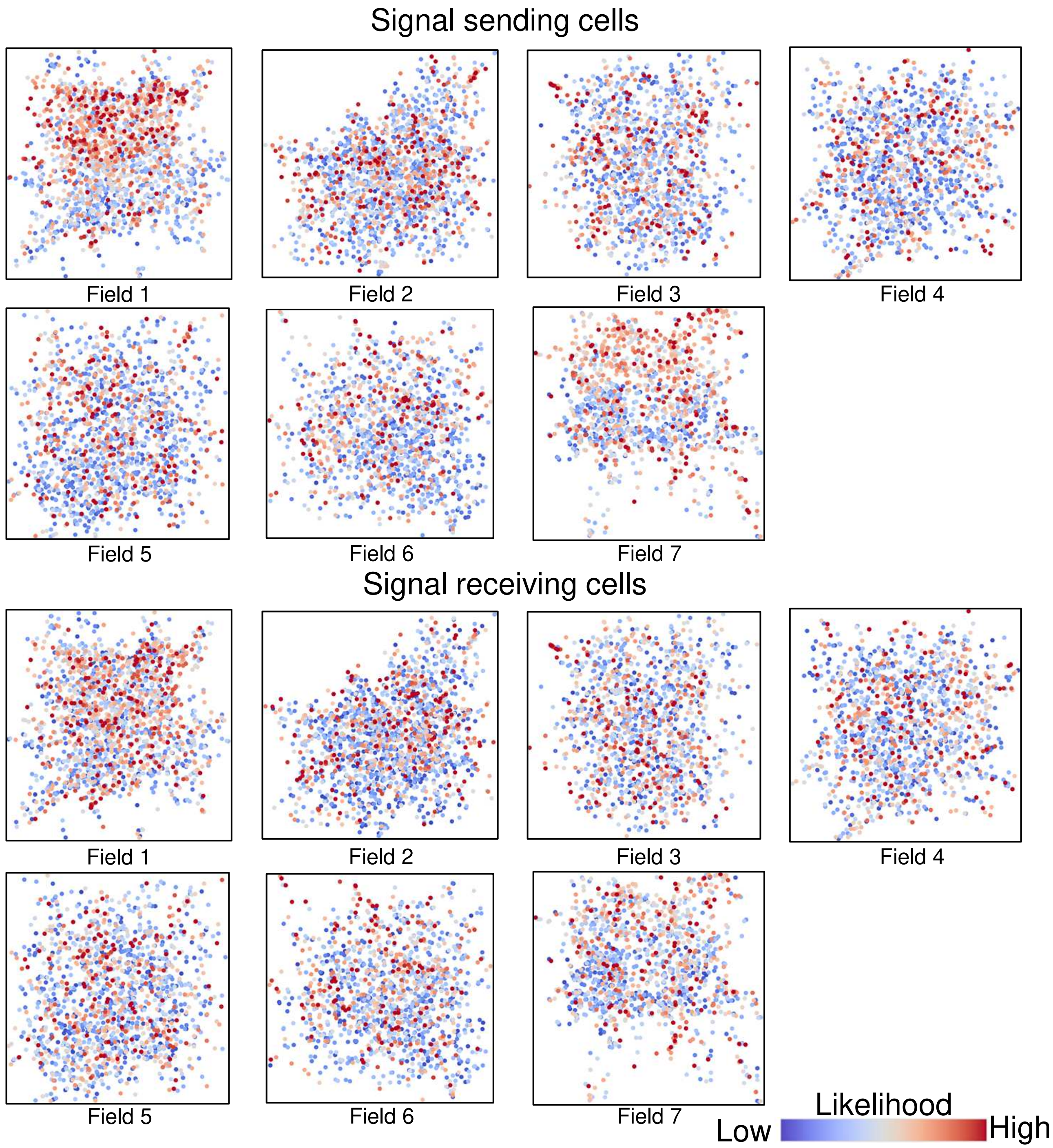

Supplementary Figure 33

The likelihood of signal sending and receiving cells of the scRNA-seq data (WT1) of mouse olfactory bulb mapped to the space of RNA seqFISH+ data.

Each dot corresponds to a single cell in the scRNA-seq data and the position is determined based on the SpaOTsc mapping of this scRNA-seq data and the reference RNA seqFISH+ data.

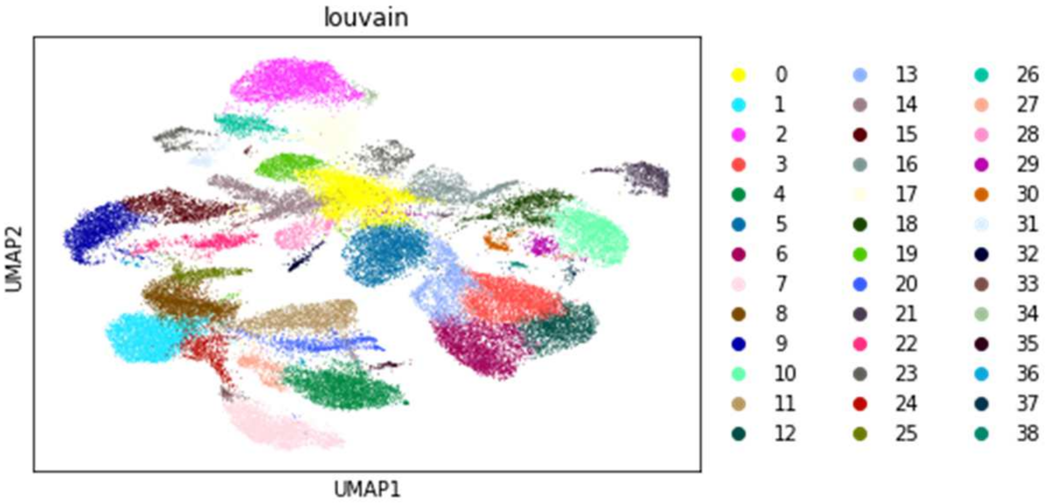

Supplementary Figure 34

Visualization of the scRNA-seq data of mouse olfactory bulb.

A low-dimensional visualization of the scRNA-seq data of mouse olfactory bulb by performing UMAP on the first 20 principle components colored by cluster identities obtained by Louvain analysis.

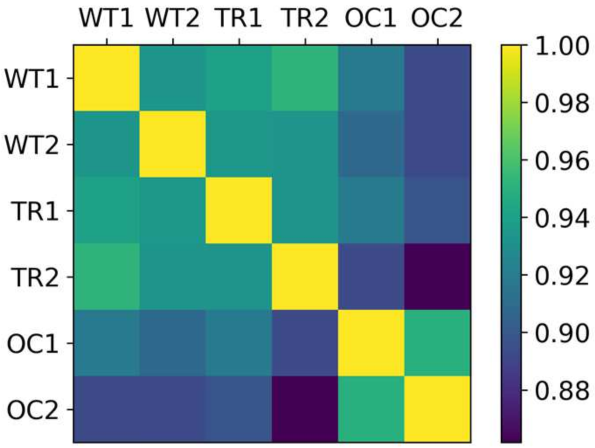

Spatial reference:
SeqFISH+ data

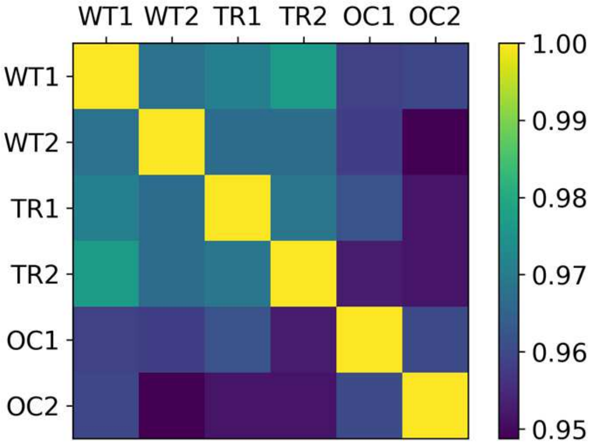

Spatial reference:
Slide-seq data

Supplementary Figure 35

Similarity of cluster level cell-cell communication patterns among the samples in scRNA-seq data of mouse olfactory bulb.

The cell-cell communications inferred for each sample in scRNA-seq data using the RNA seqFISH+ or the Slide-seq data as the spatial reference. The cell-cell communications were summarized into the 39 clusters and Pearson's correlation coefficient was used to measure the similarity between the communication patterns.
