# Supplementary material for: Inferring spatial and signaling relationships between cells from single cell transcriptomic data
Source: Nat Commun. 2020 Apr 29;11:2084. doi: 10.1038/s41467-020-15968-5 (PMC7190659; doi:10.1038/s41467-020-15968-5)
Supplement: Supplementary file 3 — Reporting Summary [file 41467_2020_15968_MOESM3_ESM.pdf]

nature research

Corresponding author(s): Qing Nie
qnie@math.uci.edu

Last updated by author(s): Mar 18, 2020

Reporting Summary

Nature Research wishes to improve the reproducibility of the work that we publish. This form provides structure for consistency and transparency in reporting. For further information on Nature Research policies, see Authors & Referees and the Editorial Policy Checklist.

Statistics

For all statistical analyses, confirm that the following items are present in the figure legend, table legend, main text, or Methods section.

n/a	Confirmed	
☐	☒	The exact sample size (*n*) for each experimental group/condition, given as a discrete number and unit of measurement
☒	☐	A statement on whether measurements were taken from distinct samples or whether the same sample was measured repeatedly
☒	☐	The statistical test(s) used AND whether they are one- or two-sided<br>*Only common tests should be described solely by name; describe more complex techniques in the Methods section.*
☒	☐	A description of all covariates tested
☒	☐	A description of any assumptions or corrections, such as tests of normality and adjustment for multiple comparisons
☒	☐	A full description of the statistical parameters including central tendency (e.g. means) or other basic estimates (e.g. regression coefficient) AND variation (e.g. standard deviation) or associated estimates of uncertainty (e.g. confidence intervals)
☒	☐	For null hypothesis testing, the test statistic (e.g. *F*, *t*, *r*) with confidence intervals, effect sizes, degrees of freedom and *P* value noted<br>*Give P values as exact values whenever suitable.*
☒	☐	For Bayesian analysis, information on the choice of priors and Markov chain Monte Carlo settings
☒	☐	For hierarchical and complex designs, identification of the appropriate level for tests and full reporting of outcomes
☐	☒	Estimates of effect sizes (e.g. Cohen's *d*, Pearson's *r*), indicating how they were calculated

Our web collection on statistics for biologists contains articles on many of the points above.

Software and code

Policy information about availability of computer code

Data collection	The STARmap software (https://github.com/weallen/STARmap) was used for acquiring the mouse visual cortex spatial data.
Data analysis	The SpaOTsc software developed in this paper is available at GitHub (https://github.com/zcang/SpaOTsc). Scanpy (v1.4.5) was used for data preprocessing for all biological systems. Seurat (v2.3.4) was used for generating similarity measurements for mouse visual cortex and mouse olfactory bulb.

For manuscripts utilizing custom algorithms or software that are central to the research but not yet described in published literature, software must be made available to editors/reviewers. We strongly encourage code deposition in a community repository (e.g. GitHub). See the Nature Research guidelines for submitting code & software for further information.

Data

Policy information about availability of data

All manuscripts must include a data availability statement. This statement should provide the following information, where applicable:

- Accession codes, unique identifiers, or web links for publicly available datasets
- A list of figures that have associated raw data
- A description of any restrictions on data availability

All results presented in this manuscript are based on publicly available datasets.
Zebrafish embryo spatial data: downloaded from (https://www.dropbox.com/s/ev78jelev0jgu5s/seurat_files_zfin.zip?dl=1) [Satija R, et al. , Nat Biotech, 2015].
Zebrafish embryo scRNA-seq data: GEO accessing codes (1) GSE66688, (2) GSE112294.
Drosophila embryo spatial and scRNA-seq data: downloaded from the Dream Single cell Transcriptomics Challenge through Synapse ID (syn15665609) [Karaiskos N, et al. Science, 2017].
Mouse visual cortex spatial data: downloaded from STARmap Resources (https://www.dropbox.com/sh/f7ebheru1lbz91s/AABYSSjSTppBmVmWl2H4s_K-a?dl=0) [Wang X, et al., Science, 2018].
Mouse visual cortex scRNA-seq data: downloaded from Allen Brain Atlas (http://celltypes.brain-map.org/api/v2/well_known_file_download/694413985) [Tasic B, et

al., Nature, 2018].
Mouse olfactory bulb Slide-seq data: downloaded from Broad Institute Single Cell Portal (https://singlecell.broadinstitute.org/single_cell/study/SCP354/slide-seq-study) [Samuel G. Rodriques et al. Science, 2019]
Mouse olfactory bulb RNA seqFISH+ data: downloaded from the Github repository (https://github.com/CaiGroup/seqFISH-PLUS) [Eng C-HL, et al. Nature, 2019]
Mouse olfactory bulb scRNA-seq data: downloaded from supplementary material of the associated publication [Tepe B, et al. Cell Reports, 2018]

Field-specific reporting

Please select the one below that is the best fit for your research. If you are not sure, read the appropriate sections before making your selection.

☒ Life sciences ☐ Behavioural & social sciences ☐ Ecological, evolutionary & environmental sciences

For a reference copy of the document with all sections, see nature.com/documents/nr-reporting-summary-flat.pdf

Life sciences study design

All studies must disclose on these points even when the disclosure is negative.

Sample size	We took the original public datasets as inputs.
Data exclusions	For both the scRNA-seq datasets of zebrafish embryo, cells from the enveloping layer were excluded using the cell identities derived in the original study that produced the datasets. These cells were not used in our study in order to be consistent with the spatial dataset where the enveloping layer is not present. For the scRNA-seq data of mouse visual cortex, the cells marked as "low quality" in the original study were excluded. Low quality cells were excluded to reduce misinterpretation of the data.
Replication	All the computational experiments were replicated three times (once on a Mac computer and twice on a desktop running Ubuntu) and all attempts at replication were successful.
Randomization	The allocation was random.
Blinding	All results are based on published data which have been studied in their original publications. Therefore, blinding from investigators is not possible when we reanalyzed the data. We have made several efforts to obtain unbiased results. Group allocation information was never provided to the computational algorithms. Hyperparameters were set to commonly used values. A wide range of values were tested for key parameters and we obtained robust results.

Reporting for specific materials, systems and methods

We require information from authors about some types of materials, experimental systems and methods used in many studies. Here, indicate whether each material, system or method listed is relevant to your study. If you are not sure if a list item applies to your research, read the appropriate section before selecting a response.

Materials & experimental systems

n/a	Involved in the study	
☒	☐	Antibodies
☒	☐	Eukaryotic cell lines
☒	☐	Palaeontology
☒	☐	Animals and other organisms
☒	☐	Human research participants
☒	☐	Clinical data

Methods

n/a	Involved in the study	
☒	☐	ChIP-seq
☒	☐	Flow cytometry
☒	☐	MRI-based neuroimaging
